# Mechanism by which a LINE protein recognizes its 3′ tail RNA

**DOI:** 10.1093/nar/gku753

**Published:** 2014-08-20

**Authors:** Yoshinori Hayashi, Masaki Kajikawa, Takuma Matsumoto, Norihiro Okada

**Affiliations:** 1 Graduate School of Bioscience and Biotechnology, Tokyo Institute of Technology, 4259-B-21 Nagatsuta-cho, Midori-ku, Yokohama, Kanagawa 226-8501, Japan; 2 Department of Life Sciences, National Cheng Kung University, Tainan 701, Taiwan; 3 Foundation for Advancement of International Science, Tsukuba 305-0821, Japan

## Abstract

LINEs mobilize their own copies via retrotransposition. LINEs can be divided into two types. One is a stringent type, which constitutes a majority of LINEs. The other is a relaxed type. To elucidate the molecular mechanism of retrotransposition, we used here two different zebrafish LINEs belonging to the stringent type. By using retrotransposition assays, we demonstrated that proteins (ORF2) encoded by an individual LINE recognize the cognate 3′ tail sequence of the LINE RNA strictly. By conducting *in vitro* binding assays with a variety of ORF2 proteins, we demonstrated that the region between the endonuclease and reverse transcriptase domains in ORF2 is the site at which the proteins bind the stem-loop structure of the 3′ tail RNA, showing that the strict recognition of the stem-loop structure by the cognate ORF2 protein is an important step in retrotransposition. This recognition can be bipartite, involving the general recognition of the stem by cTBR (conserved tail-binding region) of ORF2 and the specific recognition of the loop by vTBR (variable tail-binding region). This is the first report that clearly characterized the RNA-binding region in ORF2, providing the generality for the recognition mechanism of the RNA tail by the ORF2 protein encoded by LINEs.

## INTRODUCTION

Long interspersed nuclear elements (LINEs) or non-long terminal repeat (non-LTR) retrotransposons are transposable elements that comprise a large proportion of many eukaryotic genomes. Because their mobilization and/or amplification causes various alterations in their host genomes, LINEs have profound effects on eukaryotic genome evolution (1–6).

LINEs (∼4–7 kb) contain a 5′ untranslated region (UTR), open reading frames (ORFs, usually two, ORF1 and ORF2) and a 3′UTR. LINEs mobilize and amplify their own copies via a mechanism called retrotransposition, during which a LINE-encoded endonuclease (EN; 7) nicks a target site of the host genomic DNA, generating a 3′ hydroxyl group that is then used to prime reverse transcription of the LINE RNA using a LINE-encoded reverse transcriptase (RT; 8). This ‘target-primed reverse transcription’ is characteristic of LINE retrotransposition (9,10). The newly synthesized LINE DNA integrates into genomic DNA with the help of the host DNA repair system(s), although the mechanism of this integration is not well understood (11–13).

LINEs are classified into about 30 clades based on phylogenetic analysis of their RTs (14,15). We previously isolated two retrotransposition-competent LINEs, namely ZfL2-1 (with ORF1 and ORF2) and ZfL2-2 (only ORF2), from the zebrafish genome; these zebrafish LINEs belong to the L2 clade (16). By comparing the 3′ ‘tail’ RNA sequence between LINEs and SINEs (short interspersed nuclear elements), we discovered that LINEs and SINEs sometimes come in pairs and that retrotransposition of each SINE depends on the enzymatic machinery encoded by its cognate LINE (17). Furthermore, we have proposed that LINEs can also be classified into two groups, namely a stringent type and a relaxed type, according to the manner in which the LINE 3′ tail is recognized by the LINE enzymatic machinery (18,19). We reported that, in the case of stringent-type LINEs, a LINE shares a similar 3′ tail with its partner SINE (17). Such pairs were discovered in several clades, such as the CR1 clade (17–18,20–21), Tad1 clade (18) and RTE clade (22). These reports demonstrate that a LINE-encoded RT can recognize the 3′ tail of the LINE or its cognate SINE. Stringent-type LINEs are ubiquitous, and the number of identified stringent LINEs is increasing. Using a cell culture assay of retrotransposition, we reported that the UnaL2 LINE in the eel genome retrotransposes via recognition of the 3′ tail RNA by the encoded RT (23–25). Moreover, the importance of the 3′ tail was demonstrated using zebrafish LINEs (16). The requirement of the 3′UTR for retrotransposition was also reported for insect LINEs such as R2Bm and R2Dm (26,27) and SART1 (28). Accordingly, these LINEs are classified as the stringent type.

In contrast to stringent LINEs, mammalian L1 LINEs have no strict sequence requirement for RT recognition of the 3′ end except for the polyA tail (29–32). Consequently, L1s are classified as the relaxed type. L1s may have evolved from ancient stringent-type LINEs (18). Proteins encoded by each L1 mobilize the L1 RNA as well as other polyA-containing host RNAs via recognition of the polyA tail, which has resulted in the generation of copious processed pseudogenes (33). It is unknown, however, how L1 proteins recognize the polyA tail or how L1 RT preferentially reverse transcribes its cognate RNA (i.e. *cis-preference*: 34,35). Accordingly, it is important to understand the mechanism by which stringent LINEs are recognized by LINE proteins; this will facilitate elucidation of how stringent-type LINEs evolved to yield the relaxed type via changes in recognition of a specific structure in the 3′ tail (stringent type) that allowed recognition of the polyA tail (relaxed type).

We previously performed a retrotransposition assay using UnaL2, indicating that the retrotransposition frequencies of LINEs containing a mutated stem-loop sequence in the 3′ tail dramatically decreased (23). Structural analyses using nuclear magnetic resonance showed that the sequence within the 3′ tail of UnaL2 RNA is a critical determinant for formation of the proper stem-loop structure (36). However, it is not clear how this stem-loop structure of the 3′ tail is recognized by LINE proteins.

Here we used LINEs ZfL2-1 and ZfL2-2 and retrotransposition assays in cultured cells to develop an *in vitro* binding assay coupled with *in vitro* translation using wheat-germ extracts and quantitative polymerase chain reaction (PCR). Our results provide molecular evidence that retrotransposition depends on the recognition of each 3′ stem-loop structure by a cognate LINE protein.

## MATERIALS AND METHODS

### Oligonucleotide primers

Supplementary Table S1 lists oligonucleotide primers used in this study.

### Plasmid constructs

Plasmid DNAs used in the retrotransposition assay and those used to synthesize proteins are presented in the Supplementary material.

### Plasmid DNA preparation

Plasmid DNAs used in our transfection experiments and used to synthesize proteins were all purified using the QIAprep Spin Miniprep kit (Qiagen).

### Cell culture

HeLa-RC cells ( RC means retrotransposition competent;^16^) were grown in high-glucose Dulbecco's modified Eagle's medium (Invitrogen) in the absence of pyruvate and supplemented with 2 mM l-glutamine and 10% fetal bovine serum. All cell cultures were maintained at 37°C in a humidified atmosphere of 5% CO_2_.

### Retrotransposition assay

HeLa-RC cells (2 × 10^5^ cells/well) were seeded in six-well dishes. One day after seeding, the cells were transfected with 500 ng plasmid DNA using FuGENE6 Transfection Reagent (Roche). Cells containing the plasmid were selected with hygromycin (200 μg/ml) for 5 days. Hygromycin-resistant cells were trypsinized and reseeded into new 100-mm dishes and grown in medium with 400 μg/ml G418. In parallel, the hygromycin-resistant cells were also reseeded in a 100-mm dish and grown in medium without G418 to measure the number of viable cells. After a 10-day incubation, cell colonies were fixed with 100% ethanol and stained with 2% Giemsa solution. Retrotransposition frequency (RF) was calculated as the number of G418-resistant colonies per viable hygromycin-resistant cell.

### *In vitro* binding assay

gtZfL2-2p and the TBR and TX series of mutants were synthesized by *in vitro* translation with wheat-germ extract (Promega) according to the manufacturer's instructions. Various stem-loop RNAs were synthesized by *in vitro* transcription (TaKaRa) according to the manufacturer's instructions. First, a synthesized protein and 10 fmol of a stem-loop RNA and 2 μg of yeast tRNA were incubated for 10 min at room temperature in 20 μl of 25 mM Tris–HCl, pH 7.8, containing 200 mM NaCl and 0.1% (w/v) Tween 20 and including 150 μg protein G-coupled Dynabeads (Invitrogen). Second, the beads were washed five times with 200 μl of 25 mM Tris–HCl, pH 7.8, containing 50 mM NaCl and then collected using a magnet and resuspended in 10 μl of this same buffer. Third, the beads were incubated at 80°C for 5 min, and 1 μl was used for reverse transcription with PrimeScript RT (TaKaRa). Finally, each PCR product was subjected to quantitative RT-PCR using SYBR Premix Ex TaqII (TaKaRa).

## RESULTS

### Characterization of ZfL2-1 and ZfL2-2

**Figure 1.**
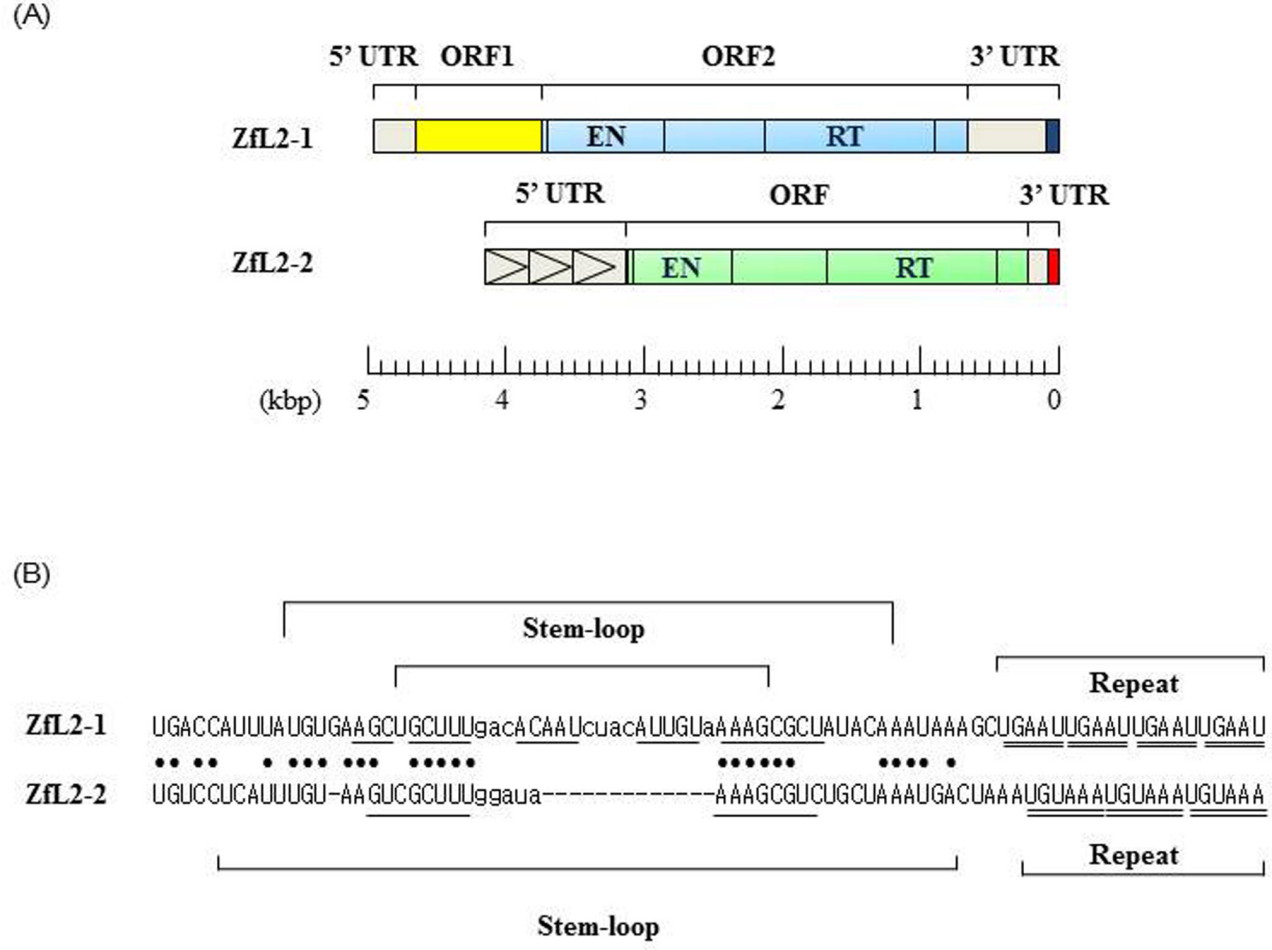
Structures of ZfL2-1 and ZfL2-2. (**A**) Schematic representation of ZfL2-1 and ZfL2-2. ZfL2-1 (5.0 kb) has two ORFs, ORF1 and ORF2. ZfL2-2 (∼4.2 kb) has one ORF. Each LINE encodes a protein containing an endonuclease domain (EN) and a RT domain. Horizontal arrowheads indicate units of the repeat sequence in the 5′UTR of ZfL2-2. Dark blue and red represent the 3′ tail in the 3′UTR of ZfL2-1 and ZfL2-2, respectively. (**B**) Nucleotides in lowercase letters denote the loop region, and the underlining indicates base-paired sequences. The upper and lower stem regions that can undergo intramolecular base pairing are underlined in each LINE. Each repeat unit is doubly underlined. Identical nucleotides are indicated by black dots.

The two zebrafish LINEs ZfL2-1 and ZfL2-2 (Figure 1A) belong to the stringent type, in which each LINE has a specific 3′ tail structure that is recognized by RT, and this is responsible for retrotransposition (16). Using HeLa cells, we previously performed retrotransposition assays for ZfL2-1 and ZfL2-2 in which the 3′UTR (containing the 3′ tail) was deleted; this was found to prohibit retrotransposition, indicating that each respective 3′ tail (Figure 1B) is important for retrotransposition (16,37).

### Each of ZfL2-1 and ZfL2-2 specifically recognizes its cognate stem-loop structure

**Figure 2.**
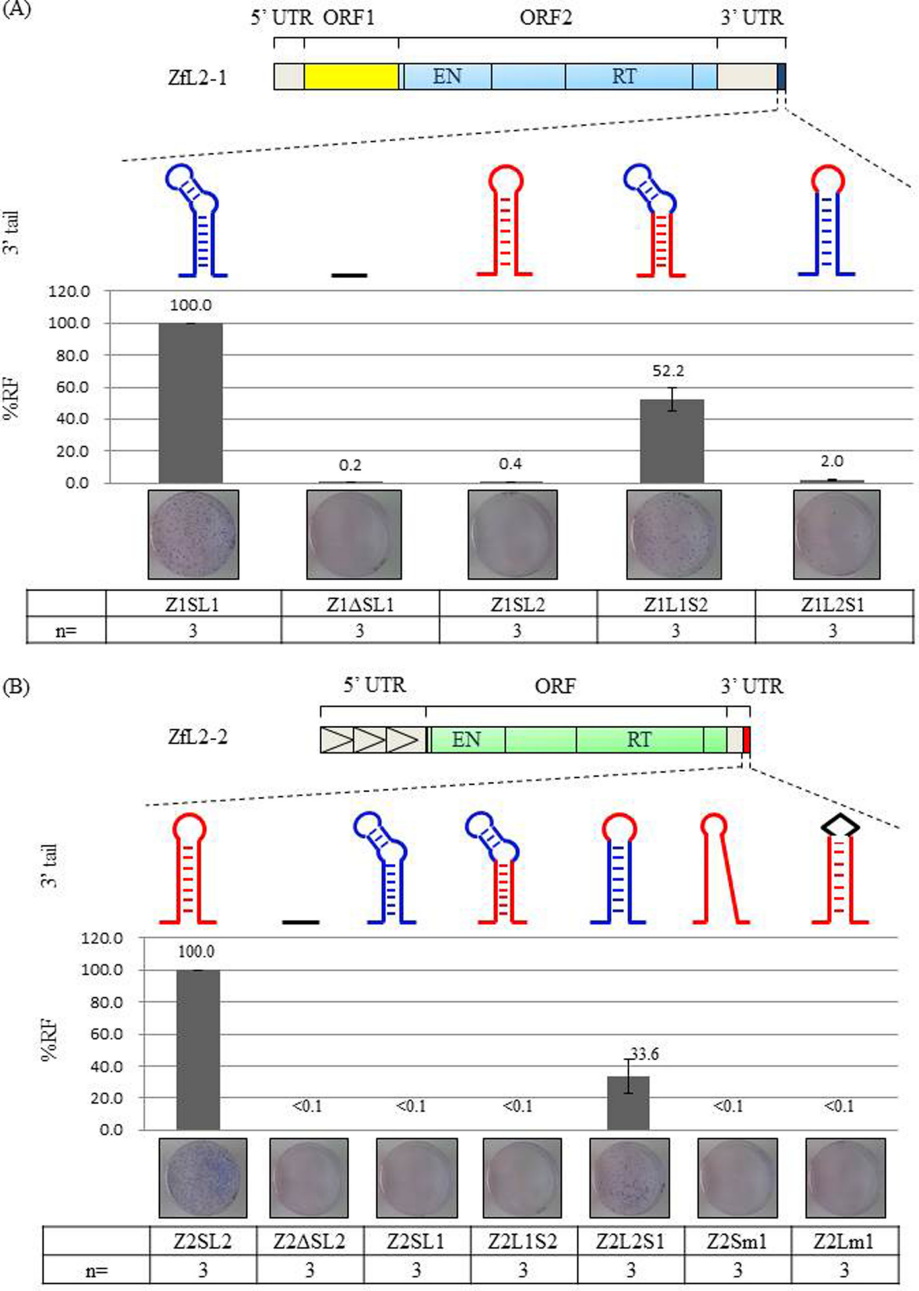
Each respective stem-loop structure of ZfL2-1 and ZfL2-2 is essential for retrotransposition. RF was calculated as described in Experimental Procedures. The %RF represents the relative RF for mutants compared with Z1SL1 or Z2SL2. Three independent experiments were performed for each construct. Schematic representations of various mutated stem-loop structures are shown. Each blue and red line denotes a sequence derived from ZfL2-1 or ZfL2-2, respectively. (**A**) Various ZfL2-1 stem-loop mutants used for the retrotransposition assay are shown. Z1SL1: ZfL2-1 having the cognate stem-loop sequence (wild type); Z1ΔSL1: ZfL2-1 lacking the cognate stem-loop sequence; Z1SL2: ZfL2-1 with the stem-loop sequence replaced with that of ZfL2-2; Z1L1S2: ZfL2-1 with stem region replaced by the cognate stem of ZfL2-2 without changing its loop region; Z1L2S1–11: ZfL2-1 with the loop region replaced by the cognate loop of ZfL2-2 without changing its stem region. (**B**) Retrotransposition assay using ZfL2-2 and its various stem-loop mutants. Z2SL2: ZfL2-2 having its cognate stem-loop sequence (wild type); Z2ΔSL2: ZfL2-2 lacking the cognate stem-loop sequence; Z2SL1: ZfL2-2 with the stem-loop sequence replaced by that of ZfL2-1; Z2L1S2: ZfL2-2 with the stem region replaced by the cognate stem of ZfL2-1 without changing its loop region; Z2L2S1: ZfL2-2 with the stem region replaced by the cognate stem of ZfL2-1 without changing its loop region; Z2Sm1: ZfL2-2 in which mutations were introduced into the stem sequence to prohibit secondary structure formation; Z2Lm1: ZfL2-2 in which mutations were introduced into the loop sequence. Data represent the mean ± SEM.

To validate the importance of the 3′ stem-loop structure in retrotransposition, we conducted retrotransposition assays using various stem-loop mutants of each LINE. The stem-loop-deleted mutants of ZfL2-1 (Z1ΔSL1; Figure 2A) and ZfL2-2 (Z2ΔSL2; Figure 2B) could not retrotranspose in HeLa cells. In addition, the stem-loop-swapped mutants of ZfL2-1 (Z1SL2; Figure 2A) and ZfL2-2 (Z2SL1; Figure 2B) also could not retrotranspose. These results showed that a specific stem-loop structure in the 3′ tail of each LINE is essential for retrotransposition. Next, we examined which region of the stem-loop structure is crucial for retrotransposition. ZfL2-1 in which its stem region was replaced by the cognate stem of ZfL2-2 without changing its loop region could retrotranspose at about half the rate of wild type (Z1L1S2; Figure 2A), whereas the RF decreased dramatically for ZfL2-1 in which its loop region was replaced by the cognate loop of ZfL2-2 with its stem region retained (Z1L2S1; Figure 2A). These results showed that retrotransposition of ZfL2-1 depends on recognition of its stem-loop structure—particularly the loop region. We performed a similar experiment with ZfL2-2; the ZfL2-2 in which the stem region was replaced by the cognate stem of ZfL2-1 without change of its loop region could retrotranspose (Z2L2S1; Figure 2B). A ZfL2-2 stem mutant having a disrupted secondary structure, however, could not retrotranspose (Z2Sm1; Figure 2B). In addition, a ZfL2-2 loop mutant could not retrotranspose (Z2Lm1; Figure 2B). These results indicated that the stem-loop structure of each respective LINE is essential for retrotransposition and that each LINE retrotransposes through the specific recognition of its loop region by the cognate RT.

### A ZfL2-2-encoded protein binds its cognate stem-loop structure

The LINE proteins ORF1p and ORF2p form a ribonucleoprotein particle (RNP) that probably is an intermediate in the retrotransposition process (38–43). The above experiments with cultured cells suggested that the stem-loop structure at the 3′ end of each LINE is crucial for retrotransposition via binding of a LINE protein(s). To validate this hypothesis, we established an *in vitro* binding assay involving a LINE protein and LINE RNA.

**Figure 3.**
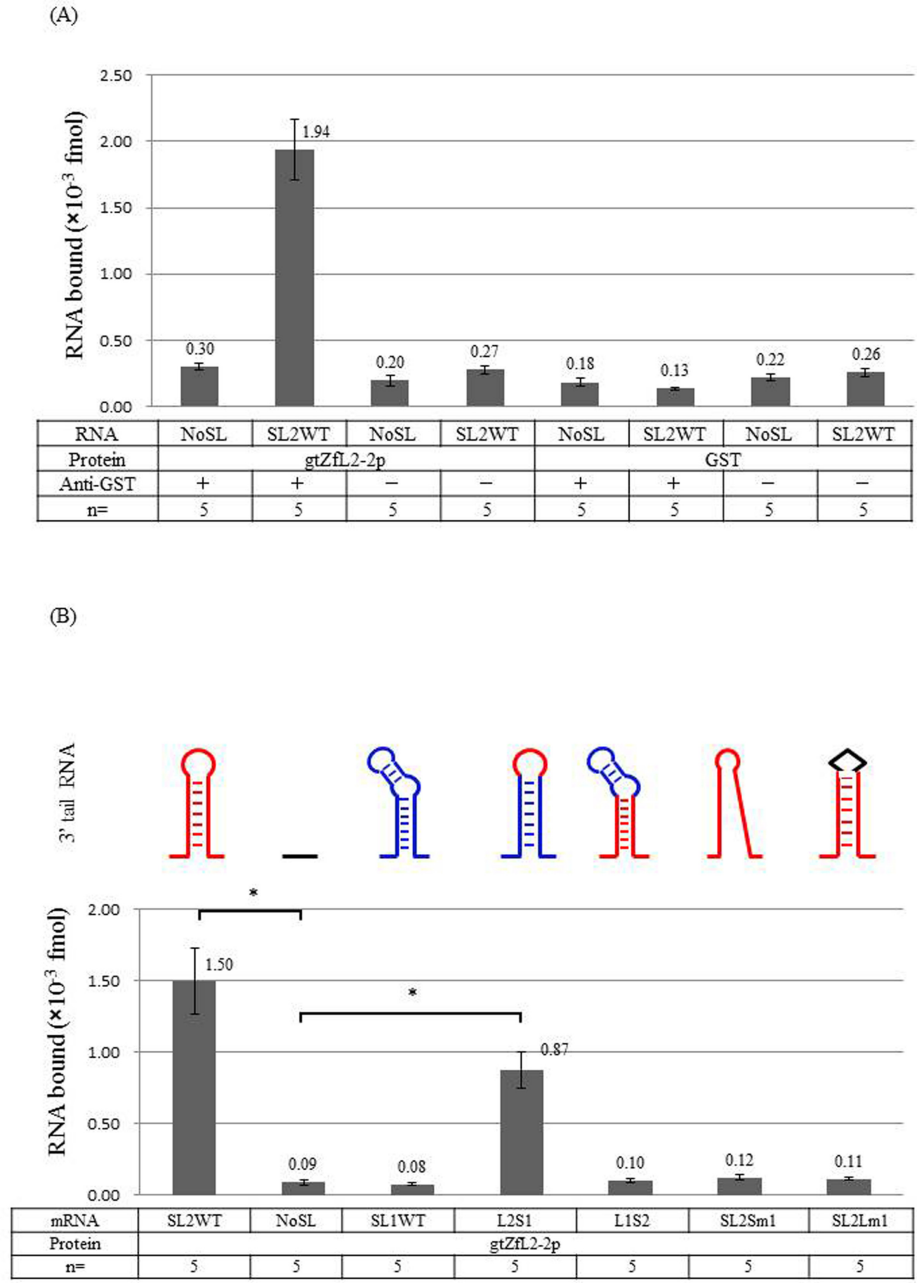
gtZfL2-2p binds to ZfL2-2 stem-loop RNA. The *in vitro* binding assay is described in Experimental Procedures. RNA binding to GST-tagged ZfL2-2 protein (gtZfL2-2p) was quantified by quantitative RT-PCR. (**A**) Validity of the *in vitro* binding assay. SL2WT: RNA including the stem-loop sequence of ZfL2-2; NoSL: RNA lacking the stem-loop of ZfL2-2. (**B**) Specificity of gtZfL2-2p binding to various stem-loop RNAs. SL2Sm1: Stem-loop RNA of ZfL2-2 whose secondary structure was perturbed by mutations; SL1WT: stem-loop RNA of ZfL2-1; L1S2: stem-loop RNA with stem region from ZfL2-2 and loop region from ZfL2-1; L2S1: stem-loop RNA with stem region from ZfL2-1 and loop region from ZfL2-2; SL2Lm1: stem-loop RNA whose loop region of ZfL2-2 was perturbed by mutations. Five independent experiments were done for each RNA. Data represent the mean ± SEM. The asterisk indicates *P* < 0.05 by Bonferroni.

Briefly, GST-tagged ZfL2-2 protein (gtZfL2-2p) and the stem-loop RNA were synthesized by *in vitro* translation and transcription, respectively. gtZfL2-2p, a protein covering the whole ORF2 containing EN and RT, represents a protein produced from a plasmid named gtZfL2-2 DNA. The *in vitro* binding assay followed by RT-PCR (Supplementary Figure S1) was used to assess stem-loop binding to the protein (Supplementary Figure S2). As shown in Figure 3A, gtZfL2-2p bound its cognate stem-loop RNA (SL2WT) but not the RNA with a deleted stem-loop (NoSL). This experiment clearly showed that gtZfL2-2p bound specifically to its cognate RNA because no binding was observed with several mutated and control RNAs (Figure 3A), validating this experimental system.

Next, we examined which region of stem-loop RNA is responsible for binding gtZfL2-2p. gtZfL2-2p could not bind the ZfL2-1 stem-loop (SL1WT; Figure 3B). However, gtZfL2-2p bound to L2S1 in which the loop was derived from ZfL2-2 and the stem region derived from ZfL2-1; in contrast, gtZfL2-2p could not bind L1S2, in which the loop region was derived from ZfL2-1 and the stem from ZfL2-2 (Figure 3B). These results showed that ZfL2-2 protein specifically recognized its cognate loop region. gtZfL2-2p could not bind to a stem mutant RNA of ZfL2-2, the secondary structure of which could not form, demonstrating that the secondary structure of the stem-loop is important for binding gtZfL2-2p (SL2Sm1; Figure 3B). In addition, gtZfL2-2p could not bind to a mutant RNA in which the loop of ZfL2-2 was replaced with a random RNA sequence (SL2Lm1; Figure 3B), and this result also confirmed that gtZfL2-2p specifically recognized the cognate loop region. The results of this binding assay demonstrated for the first time the binding of a LINE RNA to a LINE protein ORF2 *in vitro*. These results correlated exactly with those of the retrotransposition assay in cultured cells (Figure 2), indicating that we had established an *in vitro* experimental system that reflects the first recognition step of a LINE RNA by ORF2 as the initiation step of retrotransposition.

### The region between the EN domain and the RT domain of ZfL2-2 protein binds to its cognate RNA

**Figure 4.**
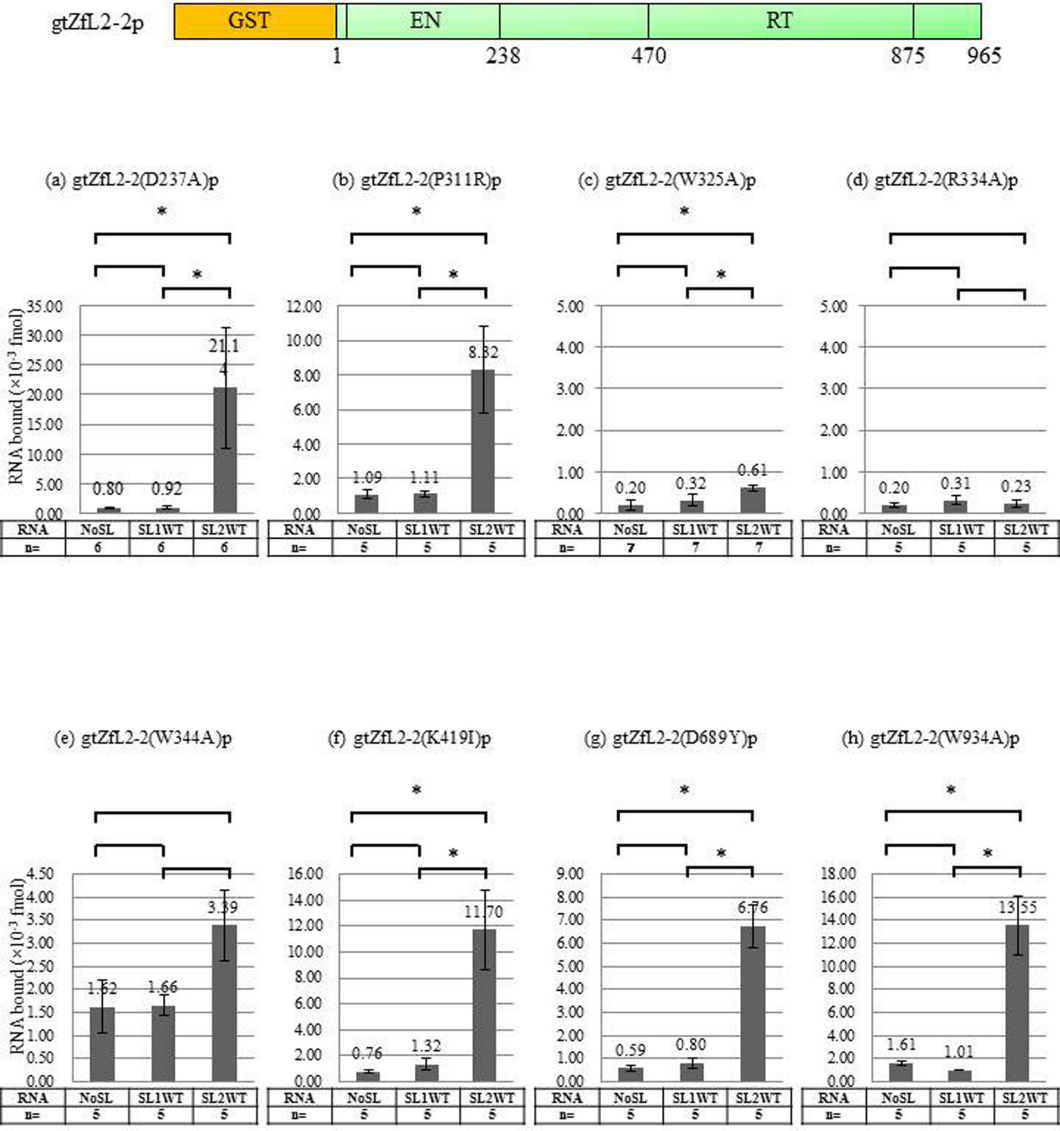
The region between the EN and RT domains of ZfL2-2 protein is responsible for binding its cognate stem-loop RNA. Binding assay with various mutated ZfL2-2p and SL2WT (or NoSL) was performed as in Figure 3. The schematic shows the domain structure of gtZfL2-2p with amino acid residue positions numbered. (**a**) gtZfL2-2(D237A)p; (**b**) gtZfL2-2(P311R)p; (**c**) gtZfL2-2(W325A)p; (**d**) gtZfL2-2(R334A)p; (**e**) gtZfL2-2(W344A)p; (**f**) gtZfL2-2(K419I)p; (**g**) gtZfL2-2(D689Y)p; (**h**) gtZfL2-2(W934A)p. At least five independent experiments were done for each protein. The asterisk indicates *P* < 0.05 by Bonferroni.

To elucidate which region of ZfL2-2 protein is responsible for binding to its cognate stem-loop RNA, we conducted binding assays using mutants of ZfL2-2 protein. We originally speculated that the region responsible for RNA binding resided within or near the RT domain. We therefore made two mutants, D689Y (D689 is essential for RT activity; 8) and W934A (W934 is conserved among members of the L2 and CR1 clades; 44). These two mutants as well as D237A (D237 is well conserved among LINEs) could bind to SL2WT (Figure 4a, g, h), suggesting that these three key residues are not critical for binding to SL2WT.

We aligned the amino acid (aa) sequences of four LINEs that are distantly related to each other (average 60% aa sequence identity in the RT domain). The alignment revealed a region abundant in basic aa residues between the EN and RT domains and that each basic residue is highly conserved among the four LINEs (Supplementary Figure S3A). Notably, these four LINEs share almost the same stem-loop sequence as ZfL2-2 (Supplementary Figure S3B), suggesting that the basic residues responsible for binding the stem-loop RNA are the same among these four LINEs. Accordingly, we introduced a mutation in each of these basic or above-mentioned conserved residues and assayed RNA-binding activity. Although proteins in which mutations were introduced outside the region could bind SL2WT (P311R, W325A and K419I; Figure 4), the binding of mutants in the basic-residue region was dramatically decreased (R334A, W344A; Figure 4 and Supplementary Figure S4). Although these results suggested that the region that includes Arg(334) and Trp(344) is responsible for ZfL2-2 stem-loop binding, the data did not exclude the possibility that the observed decreased binding capacity was due to altered three-dimensional structure of each mutant. Therefore, we assessed the RT activity of these mutants compared with wild type. The activity of gtZfL2-2(R334A)p and gtZfL2-2(W344A) as well as gtZfL2-2(R338AA339E)p was comparable to that of wild type (Supplementary Figure S5), indicating that the three-dimensional structure was not substantially perturbed in these mutants. These results confirmed that the region between the EN and RT domains in ZfL2-2 protein is responsible for binding its cognate stem-loop RNA.

### The tail-binding region in both ZfL2-1 and ZfL2-2 proteins binds the respective cognate stem-loop RNA

**Figure 5.**
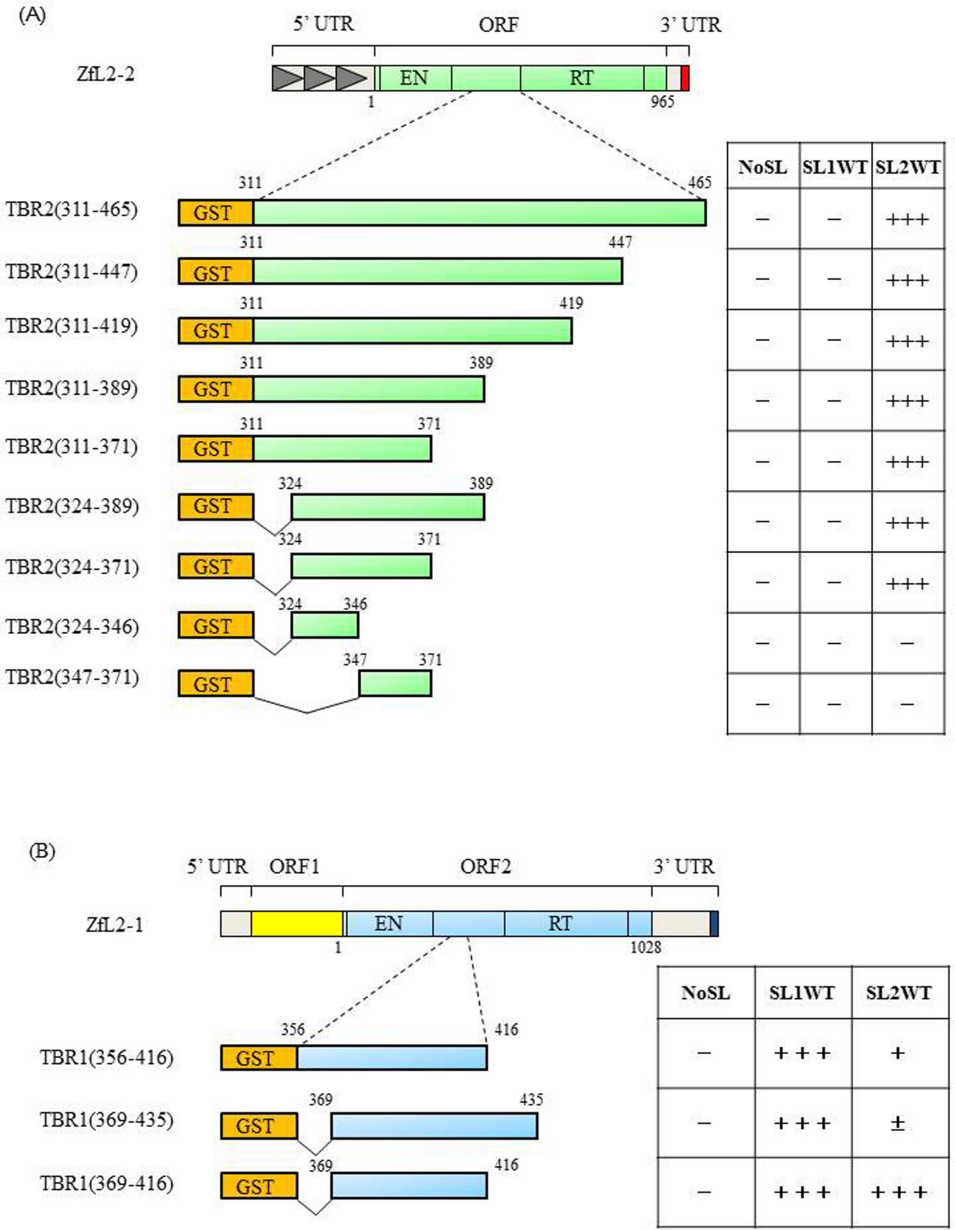
TBR1p and TBR2p bind to their respective cognate stem-loop RNA. Schematic representation of TBR corresponding to ZfL2-2 (**A**) or ZfL2-1 (**B**) protein used for the binding assay. Numbers indicate amino acid residue positions. Each (+) indicates that the protein could bind the RNA, and each (–) indicates no RNA binding, i.e. the binding was the same as that for the RNA lacking the stem-loop (NoSL). (±) indicates that there was no significant difference statistically, although the binding activity of SL2WT of TBR1(369–435)p is higher than that of NoSL. At least three independent experiments were done for each protein.

To examine in detail which region of the ZfL2-2 protein is responsible for stem-loop binding, we first constructed a plasmid encoding the region between the EN and RT domains, i.e. residues 311–465 and designated TBR2(311–465); ‘TBR’ stands for tail binding region, and ‘2’ designates ZfL2-2. We then constructed four mutants successively truncated at the 3′ end of TBR2(311–465), designated TBR2(311–447), TBR2 (311–419), TBR2 (311–389) and TBR2(311–371). We also constructed two mutants, namely TBR2(324–389), truncated from the 5′ end of TBR2(311–389), and TBR2(324–371), truncated from the 3′ end of TBR2(324–389). These seven mutant proteins could bind the ZfL2-2 stem-loop *in vitro* (Figure 5 and Supplementary Figure S6). Note that TBR2(324–371)p as well as other mutant proteins could bind SL2WT but not SL1WT (Figure 5A and Supplementary Figure S6), retaining the specificity to discriminate between SL2WT and SL1WT for binding.

TBR2(324–371) was further divided into two regions, namely TBR2(324–346) and TBR2(347–371), but neither mutant protein could bind the ZfL2-2 stem-loop (Figure 5A and Supplementary Figure S6). We thus concluded that the ∼50-residue TBR2(324–371) was a good candidate for further analysis of binding to the ZfL2-2 stem-loop RNA.

Next, we examined whether the region in ORF2 of ZfL2-1 responsible for binding to its cognate stem-loop RNA is similar to that of ZfL2-2. We constructed a plasmid encoding a region corresponding to TBR2, designated TBR1, in which 1 means ZfL2-1. TBR1(369–416)p corresponds to TBR2(324–371) (each contains 47 residues; see alignment in Supplementary Figure S3). Surprisingly, TBR1(369–416)p could bind not only to SL1WT but also to SL2WT to the same extent (Figure 5B and Supplementary Figure S7). To examine a role of the flanking amino acids in this region, we generated two mutant proteins, namely TBR1(356–416)p and TBR1(369–435)p. Interestingly, both TBR1(356–416)p and TBR1(369–435)p, each of which contains an additional N- or C-terminal region not included in TBR1(369–416)p, could specifically bind to its cognate stem-loop RNA(SL1WT), although the binding specificity of TBR1(356–416) a little bit decreases.

### Which basic residues in the TBR are responsible for binding the stem-loop RNA of each LINE?

**Figure 6.**
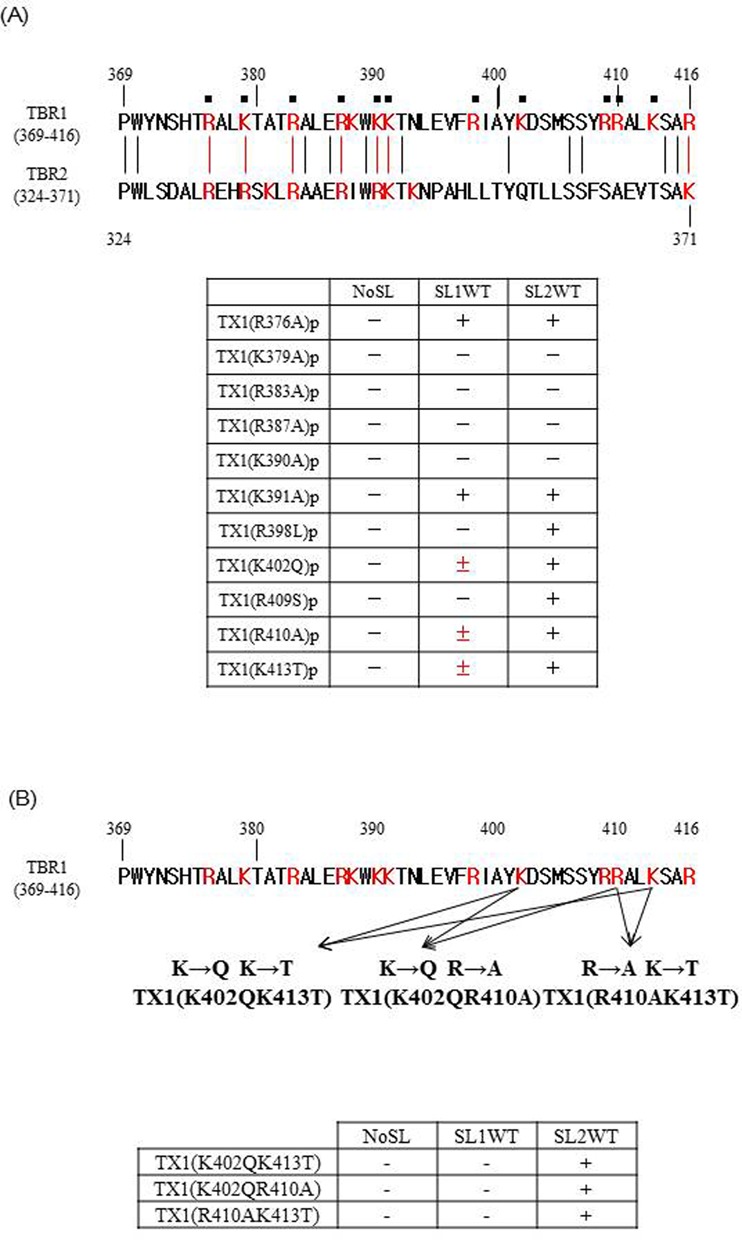
Basic residues in TBR1 (369–416) are responsible for binding the stem-loop RNA. Identical non-basic residues between sequences are indicated by black lines, and similar basic residues are indicated by red lines. Numbers indicate residue positions. Each (+) indicates that the protein can bind the RNA, and each (–) indicates no RNA binding, i.e. the binding was the same as that for the RNA lacking the stem-loop (NoSL). (±) indicates that there was no significant difference statistically, although the binding activity of SL1WT of TX1(K402Q)p, TX1(R410A)p and TX1(K413T)p is higher than that of NoSL. (**A**) Binding assay using TBR1(369–416) containing mutated basic residues common to TBR1 and TBR2. TBR1(369–416) in ZfL2-1 and TBR2(324–371) in ZfL2-2 are aligned. Binding assay using TBR1(369–416) containing mutated basic residues specific to TBR1. (**B**) Binding assay with three mutant proteins, namely TX1(K402QK413T)p, TX1(K402QR410A)p and TX1(R410AK413T)p. Red characters indicate basic residues. Five independent experiments were done for each protein.

To examine which basic residues in TBR1(369–416) are responsible for binding to each stem-loop RNA, we created the point mutant R376A in TBR1(369–416) to yield TBR1(369–416)(R376A), henceforth called TX1(R376A); R376 is common to both TBR1 and TBR2. Similarly, we made five additional mutant proteins, namely TX1(K379A)p, TX1(R383A)p, TX1(R387A)p, TX1(K390A)p and TX1(K391A)p. Among all six mutants, four—TX1(K379A)p, TX1(R383A)p, TX1(R387A)p and TX1(K390A)p—could not bind both SL1WT and SL2WT (Figure 6A and Supplementary Figure S8), indicating that basic residues K379, R383, R387 and K390 contribute to binding of both stem-loop RNAs. For TBR1, we similarly introduced a point mutation at each of five basic residues, resulting in the mutants TX1(R398L)p, TX1(K402Q)p, TX1(R409S)p, TX1(R410A)p and TX1(K413T)p.

All five of these mutants could bind to SL2WT. However, mutants, namely TX1(R398L)p and TX1(R409S)p, could not bind to SL1WT in spite of its binding to SL2WT (Figure 6A and Supplementary Figure S9), indicating that basic residues R398 and R409 contribute to specific binding of ZfL2-1 stem-loop RNA. Although the binding activity of SL1WT of three mutants, namely TX1(K402A)p, TX1(R410A)p, TX1(K4013T)p, is higher than that of NoSL, there was no significant difference statistically. To elucidate a role of these basic amino acids in the specific binding of ZfL2-1 stem-loop RNA, we made three mutants in each of which two point mutations among these three basic amino acids were introduced, namely TX1(R410AK413A)p, TX1(K402AK413A)p and TX1(K402AR410A)p (Figure 6B). Although these three mutants could bind to SL2WT, these could not bind to SL1WT (Figure 6B and Supplementary Figure S10). These results indicated that the four basic residues in these two TBRs (Figure 6A) are important for binding both to the stem-loop RNA of ZfL2-1 and ZfL2-2, and that five basic residues specific in TBR1 (Figure 6A) are important for binding to the cognate ZfL2-1 stem-loop RNA.

Finally, we performed the retrotransposition assay using a LINE with a mutated TBR. As explained above, the mutant gtZfL2-2(R334A)p produced from the mutated TBR could not bind to ZfL2-2 stem-loop RNA (Figure 4). The other mutant, gtZfL2-2(W325A)p, produced from the mutated TBR could bind this RNA (Figure 4). To examine whether the binding ability of the TBR correlated with the potential for retrotransposition, we constructed two plasmids encoding gtZfL2-2(W325A)p and gtZfL2-2(R334A)p. As expected, when retrotransposition assays were carried out with these two LINEs, the LINE encoding gtZfL2-2(W325A)p could retrotranspose, but the LINE encoding gtZfL2-2(R334A)p could not (Supplementary Figure S11). This provided crucial evidence that certain basic residues in the TBR of zebrafish LINEs responsible for binding the cognate stem-loop RNA actually contribute to retrotransposition.

## DISCUSSION

### TBR is conserved among several LINEs in the L2 clade

As shown in Figure 2, the loop region of the 3′ tail is essential for retrotransposition of ZfL2-1 and ZfL2-2. Although we observed retrotransposition of a ZfL2-1 mutant (Z1L1S2) in which the stem region was replaced by the stem of ZfL2-2 and of a ZfL2-2 mutant (Z2L2S1) in which the stem region was replaced by the stem of ZfL2-1, the stem mutant Z2Sm1 having a disrupted secondary structure could not retrotranspose (Figure 2). Furthermore, we previously showed that the RF of UnaL2 mutants decreased dramatically when stem sequences were changed—even with consideration of minimizing effects on the loop structure (23). In addition, Nomura *et al*. found that a single bulged nucleotide residing in the stem region of the 3′ tail of UnaL2 is requisite for efficient retrotransposition (36). All these results suggest that the stem region of the 3′ tail is also important for retrotransposition.

It has long been speculated that the region in ORF2 for binding its cognate 3′ tail resides in RT domain. Therefore, it was a surprising discovery that the N-terminus region of TERT (telomerase RT), separable from the RT motifs, is responsible for the interaction between TERT and telomerase RNA (45,46). Furthermore more recently, Eickbush's group indicated that the region responsible for 3′ tail RNA-binding resided not in RT domain but upstream of RT by using R2 LINE (47).

**Figure 7.**
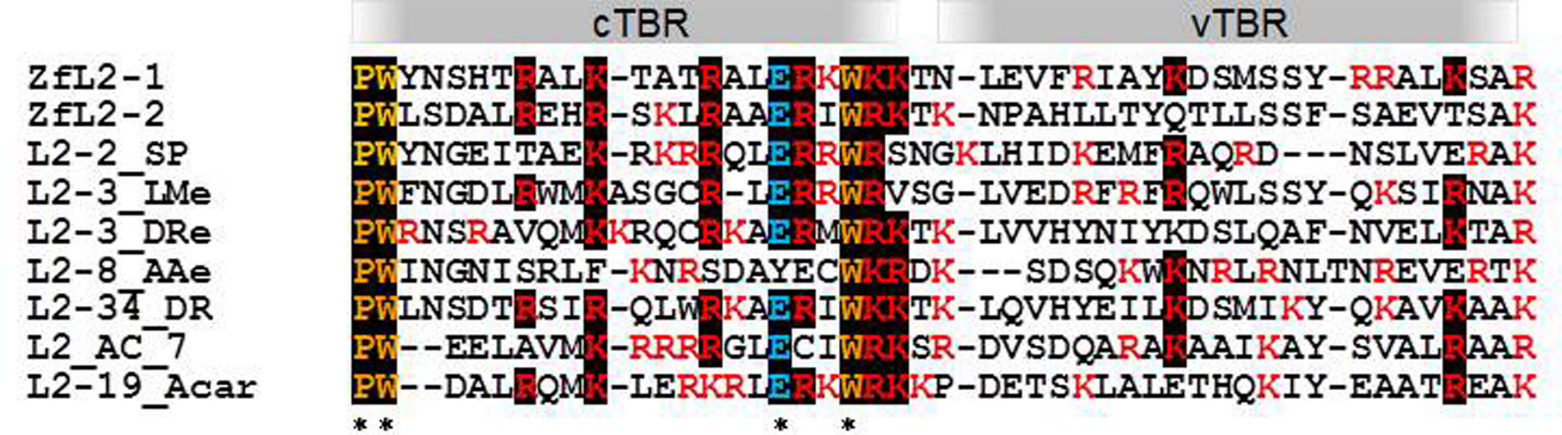
Amino acid sequence alignment of cTBR and vTBR among LINEs of the L2 clade. Residues highlighted in black are conserved among these LINEs. Basic residues are shown in red. ZfL2-1 (accession number 211149) and ZfL2-2 (accession number 211150): zebrafish LINEs used in this study; L2–2_SP: non-LTR retrotransposon from sea urchin; L2–3_LMe: coelacanth non-LTR retrotransposon (consensus sequence); L2–3_DRe: non-LTR retrotransposon from *Danio rerio* (consensus sequence); L2–8_AAe: non-LTR retrotransposon from *Aedes aegypti*; L2–34_DR: non-LTR retrotransposon from *D. rerio* (consensus sequence); L2_AC_7: non-LTR retrotransposon from *Anolis carolinensis* (consensus sequence); L2–19_Acar; non-LTR retrotransposon from *A. carolinensis* (consensus sequence). Protein sequences can be accessed from Repbase (56) at http://www.girinst.org/repbase/.

Comparison of the RNA-binding region of TBR1(369–391)p of ZfL2-1 (23 residues) and the corresponding region of TBR2(324–346)p of ZfL2-2 (23 residues) revealed that they can be closely aligned (Figure 7). Interestingly, this region is conserved among several LINEs that are distantly related within the L2 clade (Figure 7). We speculate that this region might be responsible for recognizing a structure common to L2-clade LINEs. We designated this conserved region as cTBR. As shown in Figure 6A, when we introduced a point mutation into a conserved basic residue in cTBR, four such mutant proteins could not bind to both stem-loop RNAs of zebrafish LINEs. Therefore it is reasonable to consider that these four basic residues are important for recognition of the common structure of both zebrafish LINEs.

Additionally, several non-basic residues such as Pro(324), Trp(325), Glu(341) and Trp(344) in cTBR of TBR2 are well conserved among members of the L2 clade (asterisk in Figure 7). Although the binding of mutant gtZfL2-2(W325A)p to its cognate stem-loop RNA was similar to that of wild type, mutant gtZfL2-2(W344A)p could not bind the RNA (Figure 4). These data suggest that, in addition to certain basic residues, certain conserved residues in cTBR are also involved in RNA binding possibly via maintenance of three-dimentional structure of cTBR. By contrast, the region corresponding to TBR1(392–416)p (with 25 residues juxtaposed to TBR1(369–391)p) explained above as well as the region corresponding to TBR2(347–371) (with 25 residues) is less conserved among several L2-clade LINEs (Figure 7; we designated this region as vTBR, see below). Interestingly, as shown in Figure 6A, among five mutants in which a mutation was introduced at a basic residue residing in vTBR, all could bind to its non-cognate stem-loop RNA but could not bind to its cognate stem-loop RNA. This result indicates that these five basic residues are responsible for specific binding to the loop region of the cognate 3′ tail. It is reasonable to speculate that certain residues in vTBRs have evolved to yield a TBR capable of specifically binding its own 3′ tail because this region is not well conserved among several LINEs of the L2 clade.

**Figure 8.**
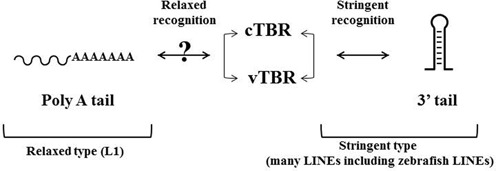
A schema of evolutionary processes by which two types of LINEs have been established via interactions between ORF2 and 3′ tail RNA. An arrow with two arrowheads indicates the interaction of concerted evolution to generate one of two types of LINEs during evolution.

In summary, we hypothesize that the cTBR is responsible for recognition of the stem structure, whereas other regions including vTBR are responsible for specific recognition of the loop structure, the molecular mechanism of which varies between LINEs. Figure 8 represents a schema showing how a LINE evolved to be a stringent-type or a relaxed-type with concerted changes between the TBR (cTBR plus vTBR) and the 3′tail RNA (regarding the relationship with a relaxed type such as L1, see Discussion).

### L1 is on the way of complete relaxation?

We hypothesize that the relaxed type was generated from the stringent type via loss of stringency during evolution (18,48). Hence, L1, a representative relaxed-type LINE, can recognize a huge number of polyA host RNAs, retrotransposition of which has created >8000 retropseudogenes in the human genome (49–55). Nevertheless, L1 preferentially mobilizes L1 RNA over cellular RNAs (‘*cis-preference*’; 34,35)^.^ The mechanism underlying this *cis-preference* for L1s is unknown. When L1 RNPs are purified from HeLa cells transfected with L1-encoding plasmid, they include not only the cognate L1 RNA but also host RNAs (35,55). When these investigators performed a LEAP (L1 Element Amplification Protocol) assay using these L1 RNPs, they discovered that L1 RNA, among the many cellular mRNAs included in RNPs, was specifically reverse-transcribed by L1 RT. These results demonstrate *cis-preference* *in vitro* (35). Although these data have not been completely confirmed, they provide a possibility that L1 RNPs specifically recognize their cognate RNAs (35,55). In another words, L1s may continue their evolution toward a more relaxed-type LINE. Our data provide an example of how LINE stringency can be altered via concerted changes of residues in the cTBR/vTBR and nucleotides in the cognate RNA, suggesting a process by which a relaxed-type LINE can be generated from a stringent type via alteration of the cTBR and vTBR. If L1 RNAs still retain some stringency, it will be interesting to pursue how such stringency can be donated by examining the region corresponding to cTBR and vTBR in L1. Namely, elucidation of the RNA-binding region corresponding to L1 ORF2 might lead to resolution of the mechanism of *cis-preference*.

## SUPPLEMENTARY DATA

Supplementary Data are available at NAR Online.

SUPPLEMENTARY DATA
