# Supplementary material for: Mechanism by which a LINE protein recognizes its 3′ tail RNA
Source: Nucleic Acids Res. 2014 Aug 20;42(16):10605–17. doi: 10.1093/nar/gku753 (PMC4176376; doi:10.1093/nar/gku753)
Supplement: SUPPLEMENTARY DATA [file supp_42_16_10605__index.html]

Mechanism by which a LINE protein recognizes its 3′ tail RNA — Mechanism by which a LINE protein recognizes its 3′ tail RNA — SUPPLEMENTARY DATA 

# Mechanism by which a LINE protein recognizes its 3′ tail RNA

## SUPPLEMENTARY DATA

**Files in this Data Supplement:**

- SUPPLEMENTARY DATA
