# Supplementary material for: Mechanism by which a LINE protein recognizes its 3′ tail RNA
Source: Nucleic Acids Res. 2014 Aug 20;42(16):10605–17. doi: 10.1093/nar/gku753 (PMC4176376; doi:10.1093/nar/gku753)
Supplement: SUPPLEMENTARY DATA [file supp_gku753_nar-03347-a-2013-File002.pdf]

Supplementary information

Plasmids used in the retrotransposition assay

The following plasmids such as Z1SL1 and Z2SL2 were constructed based on information described for pBB4 and p74.18[9]. The 3' tail of ZfL2-1 was made by PCR using the oligonucleotide primers ZW1-F1 and ZW1-R1. In this reaction, the 3' region (18 nt) of one oligonucleotide was annealed to that of another, and then PCR was occurred using the remaining regions of the oligonucleotides as template. The resulting fragment was digested with BamHI, and the fragments were inserted into the BamHI site of p74.18. The resulting plasmid was termed Z1SL1. Similarly, the 3' tail of ZfL2-2 was made by PCR using the oligonucleotide primers ZW2-F1 and ZW2-R1. The resulting plasmid was termed Z2SL2.

Z1SL2 is ZfL2-1 in which its stem-loop sequence was replaced by that of ZfL2-2. This 3' tail was made by PCR using the oligonucleotides ZW2-F1 and ZW2-R1. The resulting fragment was digested with BamHI, and the fragments were inserted into the BamHI site of p74.18.

Z1L1S2 is ZfL2-1 in which its stem region was replaced by the cognate stem of ZfL2-2 without change of its loop region. This 3' tail was made by PCR using the oligonucleotides ZL1S2-F1 and ZL1S2-R1. The resulting fragment was digested with BamHI, and the fragments were inserted into the BamHI site of p74.18.

Z1L2S1 is ZfL2-1 in which its loop region was replaced by the cognate loop of ZfL2-2 with its stem region. This 3' tail was made by PCR using the oligonucleotides ZL2S1-F1 and ZL2S1-R1. The resulting fragment was digested with BamHI, and the fragments were inserted into the BamHI site of p74.18.

Z2SL1 is ZfL2-2 in which its stem-loop sequence was replaced by that of ZfL2-1. This 3' tail was made by PCR using the oligonucleotides ZW1-F1 and ZW1-R1. The resulting fragment was digested with BamHI, and the fragments were inserted into the BamHI site of pBB4.

Z2L1S2 is ZfL2-2 in which its loop region was replaced by the cognate loop of ZfL2-1 with its stem region. This 3' tail was made by PCR using the oligonucleotides ZL1S2-F1 and ZL1S2-R1. The resulting fragment was digested with BamHI, and the fragments were inserted into the BamHI site of pBB4.

Z2L2S1 is ZfL2-2 in which its stem region was replaced by the cognate stem of ZfL2-1 without change of its loop region. This 3' tail was made by PCR using the oligonucleotides ZL2S1-F1 and ZL2S1-R1. The resulting fragment was digested with BamHI, and the fragments were inserted into the BamHI site of pBB4.

Z2Sm1 is ZfL2-2 in which mutations were introduced into the stem sequence to destroy the secondary structure. This 3' tail was made by PCR using the oligonucleotides ZW2-Sm-F1 and ZW2-R1. The resulting fragment was digested with BamHI, and the

fragments were inserted into the BamHI site of pBB4.

Z2Lm1 is ZfL2-2 in which mutations were introduced into the loop sequence. This 3' tail was made by PCR using the oligonucleotides ZW2-Lm-F1 and ZW2-Lm-R1. The resulting fragment was digested with BamHI, and the fragments were inserted into the BamHI site of pBB4.

Plasmids used in the binding assay

GtZfL2-2 is composed of the entire ZfL2-2 ORF sequence that was fused to GST.

GtZfL2-2(D237A) is composed of the entire ZFL2-2 ORF sequence in which Asp at 237 was replaced with Ala that was fused to GST. This region was made by inverse PCR using the oligonucleotides Z2-D237A-F1 and Z2-D237A-R1 using gtZfL2-2 as template.

GtZfL2-2(P311R) is composed of the entire ZFL2-2 ORF sequence in which Pro at 311 was replaced with Arg that was fused to GST. This region was made by inverse PCR using the oligonucleotides Z2-P311R-F1 and Z2-P311R-R1 using gtZfL2-2 as template.

GtZfL2-2(W325A) is composed of the entire ZFL2-2 ORF sequence in which Trp at 325 was replaced with Ala that was fused to GST. This region was made by inverse PCR using the oligonucleotides Z2-W325A-F1 and Z2-W325A-R1 using gtZfL2-2 as template.

GtZfL2-2(L360QF363S) is composed of the entire ZFL2-2 ORF sequence in which Leu at 360 was replaced with Gln and Phe at 363 was replaced with Ser that was fused to GST. This region was made by inverse PCR using the oligonucleotides Z2-L360QF363S-F1 and Z2-L360QF363S-R1 using gtZfL2-2 as template.

GtZfL2-2(A370EK371E) is composed of the entire ZFL2-2 ORF sequence in which Ala at 370 was replaced with Glu and Lys at 371 was replaced with Glu that was fused to GST. This region was made by inverse PCR using the oligonucleotides Z2-L360QF363S-F1 and Z2-L360QF363S-R1 using gtZfL2-2 as template.

GtZfL2-2(F415SF416S) is composed of the entire ZFL2-2 ORF sequence in which Phe at 415 was replaced with Ser and Phe at 416 was replaced with Ser that was fused to GST. This region was made by inverse PCR using the oligonucleotides Z2-F415SF416S-F1 and Z2-F415SF416S-R1 using gtZfL2-2 as template.

GtZfL2-2(K419I) is composed of the entire ZFL2-2 ORF sequence in which Lys at 419 was replaced with Ile that was fused to GST. This region was made by inverse PCR using the oligonucleotides Z2-K419I-F1 and Z2-K419I-R1 using gtZfL2-2 as template.

GtZfL2-2(D689Y) composes of the entire ZFL2-2 ORF sequence in which Asp at 689 was replaced with Tyr that was fused to GST. This region was made by inverse PCR using the oligonucleotides Z2-D689Y-F1 and Z2-D689Y-R1 using gtZfL2-2 as

template.

GtZfL2-2(W934A) is composed of the entire ZFL2-2 ORF sequence in which Trp at 934 was replaced with Ala that was fused to GST. This region was made by inverse PCR using the oligonucleotides Z2-W934A-F1 and Z2-W934A-R1 using gtZfL2-2 as template.

GtZfL2-2(R334A) is composed of the entire ZFL2-2 ORF sequence in which Arg at 334 was replaced with Ala that was fused to GST. This region was made by inverse PCR using the oligonucleotides m33-F1 and m33-R1 using gtZfL2-2 as template.

GtZfL2-2(R338AA339E) is composed of the entire ZFL2-2 ORF sequence in which Arg at 338 was replaced with Ala and Ala at 339 was replaced with Glu that was fused to GST. This region was made by inverse PCR using the oligonucleotides m34-F1 and m34-R1 using gtZfL2-2 as template.

TBR2(311-447) is composed of the region covering residues 311–447 of ZfL2-2 that was fused to GST. This region was made by PCR using the oligonucleotides m39-F1 and m42-R2 using gtZfL2-2 as template. The PCR product was cloned into the pEU-E01G (TEV)-N2 vector digested with NotI and XhoI by using the In-Fusion HD Cloning kit w/Cloning Enhancer (TaKaRa).

TBR2(311-419) is composed of the region covering residues 311–419 of ZfL2-2 that was fused to GST. This region was made by PCR using the oligonucleotides m39-F1 and m43-R2 using gtZfL2-2 as template. The PCR product was cloned into the pEU-E01G (TEV)-N2 vector digested with NotI and XhoI by using the In-Fusion HD Cloning kit w/Cloning Enhancer (TaKaRa).

TBR2(311-371) is composed of the region covering residues 311–371 of ZfL2-2 that was fused to GST. This region was made by PCR using the oligonucleotides m39-F1 and m49andm51-R1 using gtZfL2-2 as template. The PCR product was cloned into the pEU-E01G (TEV)-N2 vector digested with NotI and XhoI using the In-Fusion HD Cloning kit w/Cloning Enhancer (TaKaRa).

TBR2(324-389) is composed of the region covering residues 324–389 of ZfL2-2 that was fused to GST. This region was made by PCR using the oligonucleotides m50andm51-F1 and m48andm50-R1 using gtZfL2-2 as template. The PCR product was cloned into the pEU-E01G (TEV)-N2 vector digested with NotI and XhoI by using the In-Fusion HD Cloning kit w/Cloning Enhancer (TaKaRa).

TBR2(324-371) is composed of the region covering residues 324–371 of ZfL2-2 that was fused to GST. This region was made by PCR using the oligonucleotides m50andm51-F1 and m49andm51-R1 using gtZfL2-2 as template. The PCR product was cloned into the pEU-E01G (TEV)-N2 vector digested with NotI and XhoI by using the In-Fusion HD Cloning kit w/Cloning Enhancer (TaKaRa).

TBR2(324-346) is composed of the region covering residues 324–346 of ZfL2-2 that

was fused to GST. This region was made by PCR using the oligonucleotides ZFGm92-F1 and ZFGm92-R1. In this reaction, the 3’ region (55 nt) of one oligonucleotide was annealed to that of another, and then PCR occurred using the remaining region of the oligonucleotides as template. The PCR product was cloned into the pEU-E01G (TEV)-N2 vector digested with NotI and XhoI by using the In-Fusion HD Cloning kit w/Cloning Enhancer (TaKaRa).

TBR2(347-371) is composed of the region covering residues 324–371 of ZfL2-2 that was fused to GST. This region was made by PCR using the oligonucleotides ZFGm93-F1 and ZFGm93-R1. In this reaction, the 3’ region (49 nt) of one oligonucleotide was annealed to that of another, and then PCR occurred using the remaining region of the oligonucleotides as template. The PCR product was cloned into the pEU-E01G (TEV)-N2 vector digested with NotI and XhoI by using the In-Fusion HD Cloning kit w/Cloning Enhancer (TaKaRa).

TBR1(356-416) is composed of the region covering residues 356–416 of ZfL2-1 that was fused to GST. This region was made by PCR using the oligonucleotides m41-F1 and m53andm55-R1 using p74.18 as template. The PCR product was cloned into the pEU-E01G (TEV)-N2 vector digested with NotI and XhoI by using the In-Fusion HD Cloning kit w/Cloning Enhancer (TaKaRa).

TBR1(369–435) is composed of the region covering residues 369–435 of ZfL2-1 that was fused to GST. This region was made by PCR using the oligonucleotides m54andm55-F1 and m52andm54-R1 using p74.18 as template. The PCR product was cloned into the pEU-E01G (TEV)-N2 vector digested with NotI and XhoI by using the In-Fusion HD Cloning kit w/Cloning Enhancer (TaKaRa).

TBR1(369-416) is composed of the region covering residues 369–416 of ZfL2-1 that was fused to GST. This region was made by PCR using the oligonucleotides m54andm55-F3 and m53andm55-R3 using m54andm55-F4 primer as template. The PCR product was cloned into the pEU-E01G (TEV)-N2 vector digested with NotI and XhoI by using the In-Fusion HD Cloning kit w/Cloning Enhancer (TaKaRa).

TX1(R376A) is composed of the region of TBR1(369–416) in which Arg at 376 was replaced with Ala that was fused to GST. This region was made by PCR using the oligonucleotides ZFGm85-F1 and ZFGm85-R1 using ZFGm85-F2 primer as template. The PCR product was cloned into the pEU-E01G (TEV)-N2 vector digested with NotI and XhoI by using the In-Fusion HD Cloning kit w/Cloning Enhancer (TaKaRa).

TX1(K379A) is composed of the region of TBR1(369–416) in which Lys at 379 was replaced with Ala that was fused to GST. This region was made by PCR using the oligonucleotides ZFGm86-F1 and ZFGm86-R1 using ZFGm86-F2 primer as template. The PCR product was cloned into the pEU-E01G (TEV)-N2 vector digested with NotI and XhoI by using the In-Fusion HD Cloning kit w/Cloning Enhancer (TaKaRa).

TX1(R383A) is composed of the region of TBR1(369–416) in which Lys at 383 was

replaced with Ala that was fused to GST. This region was made by PCR using the oligonucleotides ZFGm87-F1 and ZFGm87-R1 using ZFGm87-F2 primer as template. The PCR product was cloned into the pEU-E01G (TEV)-N2 vector digested with NotI and XhoI by using the In-Fusion HD Cloning kit w/Cloning Enhancer (TaKaRa).

TX1(R387A) is composed of the region of TBR1(369–416) in which Arg at 387 was replaced with Ala that was fused to GST. This region was made by PCR using the oligonucleotides ZFGm88-F1 and ZFGm88-R1 using ZFGm88-F2 primer as template. The PCR product was cloned into the pEU-E01G (TEV)-N2 vector digested with NotI and XhoI by using the In-Fusion HD Cloning kit w/Cloning Enhancer (TaKaRa).

TX1(K390A) is composed of the region of TBR1(369–416) in which Lys at 390 was replaced with Ala that was fused to GST. This region was made by PCR using the oligonucleotides ZFGm89-F1 and ZFGm89-R1 using ZFGm89-F2 primer as template. The PCR product was cloned into the pEU-E01G (TEV)-N2 vector digested with NotI and XhoI by using the In-Fusion HD Cloning kit w/Cloning Enhancer (TaKaRa).

TX1(K391A) is composed of the region of TBR1(369–416) in which Lys at 391 was replaced with Ala that was fused to GST. This region was made by PCR using the oligonucleotides ZFGm90-F1 and ZFGm90-R1 using ZFGm90-F2 primer as template. The PCR product was cloned into the pEU-E01G (TEV)-N2 vector digested with NotI and XhoI by using the In-Fusion HD Cloning kit w/Cloning Enhancer (TaKaRa).

TX1(R398L) is composed of the region of TBR1(369–416) in which Arg at 398 was replaced with Leu that was fused to GST. This region was made by PCR using the oligonucleotides ZFGm66-F1 and ZFGm70-R1 using ZFGm70-F2 primer as template. The PCR product was cloned into the pEU-E01G (TEV)-N2 vector digested with NotI and XhoI by using the In-Fusion HD Cloning kit w/Cloning Enhancer (TaKaRa).

TX1(K402Q) is composed of the region of TBR1(369–416) in which Lys at 402 was replaced with Gln that was fused to GST. This region was made by PCR using the oligonucleotides ZFGm66-F1 and ZFGm69-R1 using ZFGm66-F2 primer as template. The PCR product was cloned into the pEU-E01G (TEV)-N2 vector digested with NotI and XhoI by using the In-Fusion HD Cloning kit w/Cloning Enhancer (TaKaRa).

TX1(R409S) is composed of the region of TBR1(369–416) in which Arg at 409 was replaced with Ser that was fused to GST. This region was made by PCR using the oligonucleotides ZFGm66-F1 and ZFGm68-R1 using ZFGm66-F2 primer as template. The PCR product was cloned into the pEU-E01G (TEV)-N2 vector digested with NotI and XhoI by using the In-Fusion HD Cloning kit w/Cloning Enhancer (TaKaRa).

TX1(R410A) is composed of the region of TBR1(369–416) in which Arg at 410 was replaced with Ala that was fused to GST. This region was made by PCR using the oligonucleotides ZFGm66-F1 and ZFGm67-R1 using ZFGm66-F2 primer as template. The PCR product was cloned into the pEU-E01G (TEV)-N2 vector digested with NotI and XhoI by using the In-Fusion HD Cloning kit w/Cloning Enhancer (TaKaRa).

TX1(K413T) is composed of the region of TBR1(369–416) in which Lys at 413 was replaced with Thr that was fused to GST. This region was made by PCR using the oligonucleotides ZFGm66-F1 and ZFGm66-R1 using ZFGm66-F2 primer as template. The PCR product was cloned into the pEU-E01G (TEV)-N2 vector digested with NotI and XhoI by using the In-Fusion HD Cloning kit w/Cloning Enhancer (TaKaRa).

TX1(R410AK413T) is composed of the region of TBR1(369-416) in which Arg at 410 was replaced by Ala and Lys at 413 was replaced by Thr that was fused to GST. This region was made by PCR using the oligonucleotides ZFGm82-F1 and ZFGm82-R1 using ZFGm82-F2 primer as template. The PCR product was cloned into the pEU-E01G (TEV)-N2 vector digested with NotI and XhoI by using the In-Fusion HD Cloning kit w/Cloning Enhancer (TaKaRa).

TX1(K402QK413T) is composed of the region of TBR1(369-416) in which Lys at 402 was replaced by Gln and Lys at 413 was replaced by Thr that was fused to GST.. This region was made by PCR using the oligonucleotides ZFGm83-F1 and ZFGm83-R1 using ZFGm83-F2 primer as template. The PCR product was cloned into the pEU-E01G (TEV)-N2 vector digested with NotI and XhoI by using the In-Fusion HD Cloning kit w/Cloning Enhancer (TaKaRa).

TX1(K402QR410A) is composed of the region of TBR1(369-416) in which Lys at 402 was replaced by Gln and Arg at 410 was replaced by Ala that was fused to GST.. This region was made by PCR using the oligonucleotides ZFGm84-F1 and ZFGm84-R1 using ZFGm84-F2 primer as template. The PCR product was cloned into the pEU-E01G (TEV)-N2 vector digested with NotI and XhoI by using the In-Fusion HD Cloning kit w/Cloning Enhancer (TaKaRa).

Assay for reverse transcriptase activity

Reverse transcriptase activity was assessed using various mutant ZfL2-2 proteins as well as a wild-type protein as an enzyme source and NoSL as the RNA template. Various mutant proteins were synthesized by in vitro translation systems with TNT SP6 High-Yield Wheat Germ Master Mix (Promega) according to the manufacturer’s instructions. NoSL was synthesized by in vitro transcription systems (TaKaRa) according to the manufacturer’s instructions. First, the synthesized protein, 10 fmol NoSL, and 100 μM Random 6 mers were incubated for 55 min at 37°C in 20 μl solution containing 500 mM Tris-HCl (pH 7.5), 1.5 M NaCl, 20 mM $MgCl_2$, and 0.5 mM of each dNTP. Next, this solution was incubated at 80°C for 5 min, and 1 μl RT-PCR product was quantified by quantitative RT-PCR using SYBR Premix Ex TaqII (TaKaRa) according to the manufacturer’s instructions.

Supplementary Table Sequences of Oligonucleotide primers

Primers for retrotransposition assay	
ZW1-F1	ATATGGATCCTGACCATTTATGTGAAGCTGCTTTGACACAATCTACAT
ZW1-R1	ATATGGATCCATTCAATTCAATTCAATTCAGCTTTATTTGTATAGCGCTTTTACAATGTAGATTGTGTCAAAG
ZW2-F1	ATATGGATCCTGTCCTCATTTGTAAGTCGCTTTGGATAAAAGCGTCTGCTAAATGAC
ZW2-R1	ATATGGATCCTTTACATTTACATTTACATTTAGTCATTTAGCAGACGCTTTTATCC
ZL1S2-F1	ATATGGATCCTGTCCTCATTTGTAAGTCGCTTTGACACAATCTACAT
ZL1S2-R1	ATATGGATCCTTTACATTTACATTTACATTTAGTCATTTAGCAGACGCTTTTACAATGTAGATTGTGTCAAAG
ZL2S1-F1	ATATGGATCCTGACCATTTATGTGAAGCTGCTTTGGATAAAAGCGCTATACAAATAAAGC
ZL2S1-R1	ATATGGATCCATTCAATTCAATTCAATTCAGCTTTATTTGTATAGCGCTTTTATCC
ZW2-Sm-F1	ATATGGATCCTGTCCTCATTTGTAAGTAAAAAAGGATAAAAGCGTCTGCTAAATGAC
ZW2-Lm-F1	ATATGGATCCTGTCCTCATTTGTAAGTCGCTTTTTCGAAAGCGTCTGCTAAATGAC
ZW2-Lm-R1	ATATGGATCCTTTACATTTACATTTACATTTAGTCATTTAGCAGACGCTTTCGAA

Primers for binding assay	
m33-F1	GTGAGCATGCCTCAAAACTTCGGGCTGCG
m33-R1	AGTTTTGAGGCATGCTCACGGAGAGCATC
m34-F1	AACTTGCGGAAGCGGAGAGAATTTGGCGG
m34-R1	CTCCGCTTCCGCAAGTTTTGAGCGATGCTC
m39-F2	CTTCCAGGGCCTCGAGCCTCTTGCATCCAG
m38_m39-R2	AAAACTAGTGCGGCCGCTTAGGTTGCATGG
m39-F1	CTTCCAGGGCCTCGAGCCTCTTGCATCCAGGCC

m42-R1	AAAACTAGTGCGGCCGCTTAAAAAGAGGTG
m43-R1	AAAACTAGTGCGGCCGCTTATTTGGTGCAG
m49andm51-R1	AAAACTAGTGCGGCCGCTTACTTTGCAGAA
m50andm51-F1	CTTCCAGGGCCTCGAGCCCTGGCTCTCGGA
m48andm50-R1	AAAACTAGTGCGGCCGCTTAAAAAAGTAGG
m41-F1	CTTCCAGGGCCTCGAGCCCATAAAATTAAAAAAGG
m53andm55-R1	AAAACTAGTGCGGCCGCTTACCTGGCAGAT
m54andm55-F1	CTTCCAGGGCCTCGAGCCATGGTATAATAG
m52andm54-R1	AAAACTAGTGCGGCCGCTTAAAATAAAAAT
m54andm55-F3	CTTCCAGGGC CTCGAGCCAT GGTATAATAG TCATACTCGT GCGCTCAAAA CAGCAACCCG CGCCCTGGAA CGTAAATGGA
m54andm55-F4	CGCCCTGGAA CGTAAATGGA AAAAAACTAA TTTAGAGGTC TTTAGAATTG CGTACAAAGA
m53andm55-R3	AAAACTAGTG CGGCCGCTTA CCTGGCAGAT TTTAGAGCCC TCCTATAGCT GGACATACTG TCTTTGTACG CAATTCTAAA
ZFGm66-F1	CTTCCAGGGCCTCGAGCCATGGTATAATAGTCATACTCGTGCGCTCAAAACAGCAACCCGCGCCCTGGAACGTAAATGGA
ZFGm66-F2	CAGCAACCCG CGCCCTGGAA CGTAAATGGA AAAAAACTAA TTTAGAGGTC TTTAGAATTG CGTACAAAGA CAGTATGTCC
ZFGm66-R1	AAAACTAGTGCGGCCGCTTACCTGGCAGATGTTAGAGCCCTCCTATAGCTGGACATACTGTCTTTGTACGCAATTCTAAA
ZFGm67-R1	AAAACTAGTGCGGCCGCTTACCTGGCAGATTTTAGAGCCGCCCTATAGCTGGACATACTGTCTTTGTACGCAATTCTAAA
ZFGm68-R1	AAAACTAGTGCGGCCGCTTACCTGGCAGATTTTAGAGCCCTACTATAGCTGGACATACTGTCTTTGTACGCAATTCTAAA
ZFGm69-R1	AAAACTAGTGCGGCCGCTTACCTGGCAGATTTTAGAGCCCTCCTATAGCTGGACATACTGTCTTGGTACGCAATTCTAAA
ZFGm70-F2	CAGCAACCCGCGCCCTGGAACGTAAATGGAAAAAAACTAATTTAGAGGTCTTTCTAATTGCGTACAAAGACAGTATGTCC
ZFGm70-R1	AAAACTAGTGCGGCCGCTTACCTGGCAGATTTTAGAGCCCTCCTATAGCTGGACATACTGTCTTTGTACGCAATTAGAAA
ZFGm79-F1	CTTCCAGGGCCTCGAGCCCTGGCTCTCGGATGCTCTCCGTGAGCATCGCTCAAAACTTCGGGCTGCGGAGAGAAAATGGC

ZFGm79-F2	CAAAACTTCGGGCTGCGGAGAGAAAATGGCGGAAAACTAAAAATCCTGCACATAGATTAACATACAAAACTCTTCTGTCC
ZFGm79-R1	AAAACTAGTGCGGCCGCTTACTTTGCAGATTTAACCTCCCTCCTGAAAGAGGACAGAAGAGTTTTGTATGTTAATCTATG
ZFGm78-F1	CTTCCAGGGCCTCGAGCCTCTTGCATCCAGGCCAGCCCGTGCCAGTCCTCCTGCACCATGGTATAATAGTCATACTCGTG
ZFGm78-R2	AAAACTAGTGCGGCCGCTTAAAAAAGTAGGCGAGGATTAGTGGCATTGTTGATTTTCAGACGGTAATACGTCTGCCTGGC
ZFGm82-F1	CTTCCAGGGCctcgagCCATGGTATAATAGTCATACTCGTGCGCTCAAAACAGCAACCCGCGCCCTGGAACGTAAATGGA
ZFGm82-F2	CAGCAACCCGCGCCCTGGAACGTAAATGGAAAAAAACTAATTTAGAGGTCTTTAGAATTGCGTACAAAGACAGTATGTCC
ZFGm82-R1	AAAACTAGTgcggccgcTTACCTGGCAGATgTTAGAGCCgcCCTATAGCTGGACATACTGTCTTTGTACGCAATTCTAAA
ZFGm83-F1	CTTCCAGGGCctcgagCCATGGTATAATAGTCATACTCGTGCGCTCAAAACAGCAACCCGCGCCCTGGAACGTAAATGGA
ZFGm83-F2	CAGCAACCCGCGCCCTGGAACGTAAATGGAAAAAAACTAATTTAGAGGTCTTTAGAATTGCGTACcAAGACAGTATGTCC
ZFGm83-R1	AAAACTAGTgcggccgcTTACCTGGCAGATgTTAGAGCCCTCCTATAGCTGGACATACTGTCTTgGTACGCAATTCTAAA
ZFGm84-F1	CTTCCAGGGCctcgagCCATGGTATAATAGTCATACTCGTGCGCTCAAAACAGCAACCCGCGCCCTGGAACGTAAATGGA
ZFGm84-F2	CAGCAACCCGCGCCCTGGAACGTAAATGGAAAAAAACTAATTTAGAGGTCTTTAGAATTGCGTACcAAGACAGTATGTCC
ZFGm84-R1	AAAACTAGTgcggccgcTTACCTGGCAGATTTTAGAGCCgcCCTATAGCTGGACATACTGTCTTgGTACGCAATTCTAAA
ZFGm85-F1	CTTCCAGGGCCTCGAGCCATGGTATAATAGTCATACTGCTGCGCTCAAAACAGCAACCCGCGCCCTGGAACGTAAATGGA
ZFGm85-F2	CAGCAACCCGCGCCCTGGAACGTAAATGGAAAAAAACTAATTTAGAGGTCTTTAGAATTGCGTACAAAGACAGTATGTCC
ZFGm85-R1	AAAACTAGTGCGGCCGCTTACCTGGCAGATTTTAGAGCCCTCCTATAGCTGGACATACTGTCTTTGTACGCAATTCTAAA
ZFGm86-F1	CTTCCAGGGCCTCGAGCCATGGTATAATAGTCATACTCGTGCGCTCGCAACAGCAACCCGCGCCCTGGAACGTAAATGGA
ZFGm86-F2	CAGCAACCCGCGCCCTGGAACGTAAATGGAAAAAAACTAATTTAGAGGTCTTTAGAATTGCGTACAAAGACAGTATGTCC
ZFGm86-R1	AAAACTAGTGCGGCCGCTTACCTGGCAGATTTTAGAGCCCTCCTATAGCTGGACATACTGTCTTTGTACGCAATTCTAAA
ZFGm87-F1	CTTCCAGGGCCTCGAGCCATGGTATAATAGTCATACTCGTGCGCTCAAAACAGCAACCGCCGCCCTGGAACGTAAATGGA
ZFGm87-F2	CAGCAACCGCCGCCCTGGAACGTAAATGGAAAAAAACTAATTTAGAGGTCTTTAGAATTGCGTACAAAGACAGTATGTCC
ZFGm87-R1	AAAACTAGTGCGGCCGCTTACCTGGCAGATTTTAGAGCCCTCCTATAGCTGGACATACTGTCTTTGTACGCAATTCTAAA
ZFGm88-F1	CTTCCAGGGCCTCGAGCCATGGTATAATAGTCATACTCGTGCGCTCAAAACAGCAACCCGCGCCCTGGAAGCTAAATGGA

ZFGm88-F2	CAGCAACCCGCGCCCTGGAAGCTAAATGGAAAAAAACTAATTTAGAGGTCTTTAGAATTGCGTACAAAGACAGTATGTCC
ZFGm88-R1	AAAACTAGTGCGGCCGCTTACCTGGCAGATTTTAGAGCCCTCCTATAGCTGGACATACTGTCTTTGTACGCAATTCTAAA
ZFGm89-F1	CTTCCAGGGCCTCGAGCCATGGTATAATAGTCATACTCGTGCGCTCAAAACAGCAACCCGCGCCCTGGAACGTAAATGGG
ZFGm89-F2	CAGCAACCCGCGCCCTGGAACGTAAATGGGCAAAAACTAATTTAGAGGTCTTTAGAATTGCGTACAAAGACAGTATGTCC
ZFGm89-R1	AAAACTAGTGCGGCCGCTTACCTGGCAGATTTTAGAGCCCTCCTATAGCTGGACATACTGTCTTTGTACGCAATTCTAAA
ZFGm90-F1	CTTCCAGGGCCTCGAGCCATGGTATAATAGTCATACTCGTGCGCTCAAAACAGCAACCCGCGCCCTGGAACGTAAATGGA
ZFGm90-F2	CAGCAACCCGCGCCCTGGAACGTAAATGGAAAGCAACTAATTTAGAGGTCTTTAGAATTGCGTACAAAGACAGTATGTCC
ZFGm90-R1	AAAACTAGTGCGGCCGCTTACCTGGCAGATTTTAGAGCCCTCCTATAGCTGGACATACTGTCTTTGTACGCAATTCTAAA

Supplementary Figure S1 Various RNA sequences used for the in vitro binding assay.

(A) DNA sequence of NoSL. Each number indicates a position in the DNA sequence. The red lowercase letters indicate a BamHI site. The horizontal arrows indicate the primers used for quantitative RT-PCR. (B) The upper scheme represents the NoSL sequence. The lower sequences indicate various RNAs used for in the vitro binding assay. These sequences were inserted into the BamHI site in NoSL.

Supplementary Figure S2 Experimental procedure for the binding assay.

(A) Schematic representation of gtZfL2-2. GST, glutathione S-transferase; EN, endonuclease; RT, reverse transcriptase; Amp, ampicillin. (B) Experimental procedure of binding assay.

Supplementary Figure S3 Alignment of four LINEs with similar stem-loop sequences.

(A) EN indicates the endonuclease domain, and RT indicates the reverse transcriptase domain. The horizontal line indicates that the region corresponding to TBR1(356-368) is abundant in basic residues (see Discussion). Residues highlighted in black represent conserved amino acids among these LINEs. ZfL2-1 (Accession No. AB211149) and ZfL2-2 (Accession No. AB211150), zebrafish LINEs used in this study; UnaL2, non-LTR retrotransposons in the eel genome (Aja6-15; Accession No. AB179624); GaL2-2 (consensus), this LINE was from *Gasterosteus aculeatus*. SsL2 (consensus), this LINE was from *Salmo salar* (consensus) (B) Alignment of stem-loop sequences of four LINEs. Black highlighting denotes ribonucleotides conserved among the four LINEs. Lowercase letters indicate the loop region.

Supplementary Figure S4 The region between the EN and RT domains of ZfL2-2 is responsible for binding to its cognate stem-loop RNA.

Binding assay was performed with various mutated ZfL2-2p and SL2WT (or NoSL). The upper scheme shows structures of the protein-coding sequence of gtZfL2-2p. Numbers indicate amino acid positions of gtZfL2-2p. SL2WT, RNA including the stem-loop RNA of ZfL2-2; NoSL, RNA with the deleted stem-loop sequence of ZfL2-2. (a) gtZfL2-2(R338AA339E)p; (b) gtZfL2-2(F3415SF416S)p. At least three independent experiments were done for each protein. Data represent the mean ± SEM. The asterisk indicates $P<0.05$ by Bonferroni.

Supplementary Figure S5 Reverse transcriptase activity of various mutant proteins.

The experimental procedure for the reverse transcriptase activity is described in Supplemental Methods. WT, gtZfL2-2p; GST, GST protein only. GtZfL2-2(R334A)p, gtZfL2-2(R338AA339E)p, gtZfL2-2(W344A)p, gtZfL2-2(L360QF363S)p, gtZfL2-2(A370EK371E)p, and gtZfL2-2(D689Y)p are described in supplemental information.

Three independent experiments were done for each protein. Data represent the mean ± SEM.

Supplementary Figure S6 TBR2p binds to its cognate stem-loop RNA.

Binding assay was performed with the region corresponding to TBR2. SL2WT, RNA including the stem-loop RNA of ZfL2-2; SL1WT, RNA including the stem-loop RNA of ZfL2-1; NoSL, RNA with the deleted stem-loop sequence of ZfL2-2. TBR2(311–465) is composed of the region covering residues 311–465 that was fused to GST; TBR2(311–447) is composed of the region covering residues 311–447 that was fused to GST; TBR2(311–419) is composed of the region covering residues 311–419 that was fused to GST; TBR2(311–389) is composed of the region covering residues 311–389 that was fused to GST; TBR2(311–371) is composed of the region covering residues 311–371 that was fused to GST; TBR2(324–389) is composed of the region covering residues 324–389 that was fused to GST; TBR2(324–371) is composed of the region covering residues 324–371 that was fused to GST; TBR2(324–346) is composed of the region covering residues 324–346 that was fused to GST; TBR2(347–371) is composed of the region covering residues 347–371 that was fused to GST. At least three independent experiments were done for each protein. Data represent the mean ± SEM. The asterisk indicates $P<0.05$ by Bonferroni.

Supplementary Figure S7 TBR1p binds to its cognate stem-loop RNA.

Binding assay was performed with the region corresponding to TBR1. SL2WT, RNA including the stem-loop RNA of ZfL2-2; SL1WT, RNA including the stem-loop RNA of ZfL2-1; NoSL, RNA with the deleted stem-loop sequence of ZfL2-2. TBR1(356–416) is composed of the region covering residues 356–416 that was fused to GST; TBR1(369–435) is composed of the region covering residues 369–435 that was fused to GST; TBR1(369–416) is composed of the region covering residues 369–416 that was fused to GST. At least five independent experiments were done for each protein. Data represent the mean ± SEM. The asterisk indicates $P<0.05$ by Bonferroni.

Supplementary Figure S8 Basic residues common to TBR1 and TBR2 in TBR1(369–416) are responsible for binding to SL1WT and SL2WT.

Binding assay using TBR1(369–416) with mutated basic residues common to TBR1 and TBR2. SL2WT, RNA including the stem-loop RNA of ZfL2-2; SL1WT, RNA including the stem-loop RNA of ZfL2-1; NoSL, RNA with the deleted stem-loop sequence of ZfL2-2. TX1(R376A)p indicates that this protein is composed of the region of TBR1(369–416) in which Arg at 376 was replaced by Ala that was fused to GST; TX1(K379A)p indicates that this protein is composed of the region of TBR1(369–416) in which Lys at 379 was replaced by Ala that was

fused to GST; TX1(R383A)p indicates that this protein is composed of the region of TBR1(369–416) in which Arg at 383 was replaced by Ala that was fused to GST; TX1(R387A)p indicates that this protein is composed of the region of TBR1(369–416) in which Arg at 387 was replaced by Ala; TX1(R390A)p indicates that this protein is composed of the region of TBR1(369–416) in which Arg at 390 was replaced by Ala that was fused to GST; TX1(K391A)p indicates that this protein is composed of the region of TBR1(369–416) in which Lys at 391 was replaced by Ala that was fused to GST. At least three independent experiments were done for each protein. Data represent the mean ± SEM. The asterisk indicates $P<0.05$ by Bonferroni.

Supplementary Figure S9 Basic residues specific to TBR1 in TBR1(369–416) are responsible for binding to its cognate stem-loop RNA.

Binding assay using TBR1(369–416) with mutated basic residues specific to TBR1. SL2WT, RNA including the stem-loop RNA of ZfL2-2; SL1WT, RNA including the stem-loop RNA of ZfL2-1; NoSL, RNA with the deleted stem-loop sequence of ZfL2-2. TX1(R398L)p indicates that this protein is composed of the region of TBR1(369–416) in which Arg at 398 was replaced by Leu that was fused to GST; TX1(K402Q)p indicates that this protein is composed of the region of TBR1(369–416) in which Lys at 402 was replaced by Gln that was fused to GST; TX1(R409Q)p indicates that this protein is composed of the region of TBR1(369–416) in which Arg at 409 was replaced by Gln that was fused to GST; TX1(R410A)p indicates that this protein is composed of the region of TBR1(369–416) in which Arg at 410 was replaced by Ala that was fused to GST; TX1(K413T)p indicates that this protein is composed of the region of TBR1(369–416) in which Lys at 413 was replaced with Thr that was fused to GST. At least three independent experiments were done for each protein. Data represent the mean ± SEM. The asterisk indicates $P<0.05$ by Bonferroni.

Supplementary Figure S10 TBR2(324–371) with five residues specific to TBR1(369–416) inserted into the corresponding to positions can bind to the SL1WT.

Binding assay with three mutant proteins, namely TX1(R410AK413T)p, TX1(K402QK413T)p and TX1(K402QR410A)p. TX1(R410AK413T)p indicates that this protein is composed of the region of TBR1(369–416) in which Arg at 410 was replaced by Ala and Lys at 413 was replaced by Thr that was fused to GST; TX1(K402QK413T)p indicates that this protein is composed of the region of TBR1(369–416) in which Lys at 402 was replaced by Gln and Lys at 413 ware replaced by Thr that was fused to GST; TX1(K402QR410A)p indicates that this protein is composed of the region of TBR1(369–416) in which Lys at 402 was replaced by Gln and Arg at 410 was replaced by Ala that was fused to GST. At least five independent

experiments were done for each protein. Data represent the mean ± SEM. The asterisk indicates P<0.05 by Bonferroni.

Supplementary Figure S11 LINE with mutated basic residues responsible for binding to its own stem-loop RNA cannot retrotranspose.

Retrotransposition assay using a LINE with a mutated TBR. Retrotransposition frequency (RF) was calculated as described in METHODS. %RF represents the relative value of RF for mutants compared with Z2SL2. Three independent experiments were performed for each construct. Upper, examples of plates containing G418^R colonies. Z2SL2, ZfL2-2 having its cognate stem-loop sequence (wild type); Z2ΔSL2, ZfL2-2 with its cognate stem-loop sequence deleted; Z2m(W325A), ZfL2-2 with a mutation at position 325, which changed Trp to Ala; Z2m(R334A), ZfL2-2 with a mutation at 334, which changed Arg to Ala. Lower, graph shows the RF values for each construct. Three independent experiments were done for each protein. Data represent the mean ± SEM.

(A)

NoSL

```
1
GGACTTTCCACACCCTAACTGACACACATTCCACAGCTGATCGATACCGTAAGCCGACACGTGCAA

                                         BamHI
TCCATATGACTAGTAGATCCTCTAGAGTCGAGGCCGGCAAGGCCggatccAGACATGATAAGATAC

ATTGATGAGTTTGGACAAACCACAACTAGAATGCAGTGAAAAAAATGCTTTATTTGTGAAATTTGT

                                             249
GATGCTATTGCTTTATTTGTAACCATTATAAGCTGCAATAAACAAGTTAAC
```

(B)

NoSL

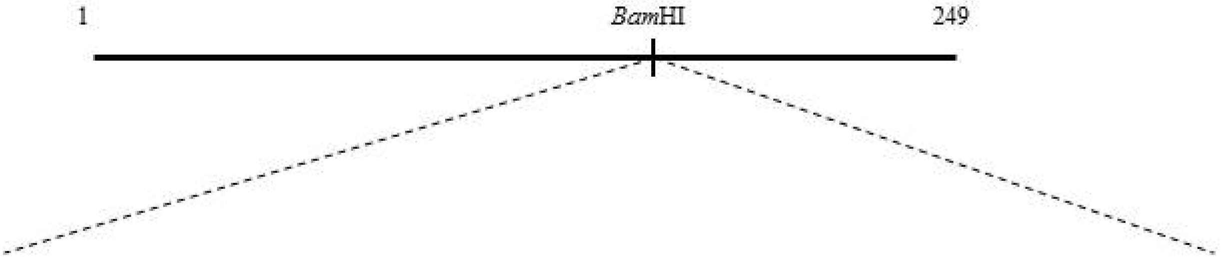


```
SL2WT   TGTCCTCATTTGT-AAGTCGCTTTggata-------------AAAGCGTCTGCTAAATGACTAAATGTAAATGTAAATGTAAA
SL1WT   TGACCATTTATGTGAAGCTGCTTTgacacaatctacattgtaAAAGCGCTATACAAATAAAGCTGAATTGAATTGAATTGAAT
SL2Sm1  TGTCCTCATTTGT-AAGTaaaaaaggata-------------AAAGCGTCTGCTAAATGACTAAATGTAAATGTAAATGTAAA
L1S2    TGTCCTCATTTGT-AAGTCGCTTTgacacaatctacattgtaAAAGCGTCTGCTAAATGACTAAATGTAAATGTAAATGTAAA
L2S1    TGACCATTTATGTGAAGCTGCTTTggata-------------AAAGCGCTATACAAATAAAGCTGAATTGAATTGAATTGAAT
SL2Lm1  TGTCCTCATTTGT-AAGTCGCTTTttcg--------------AAAGCGTCTGCTAAATGACTAAATGTAAATGTAAATGTAAA
NoSL    -----------------------------------------------------------------------------------
```

(A)

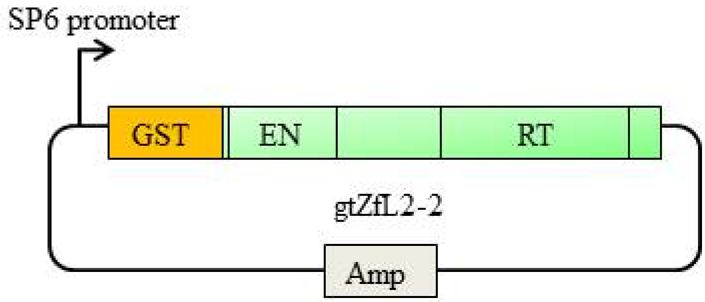
SP6 promoter
GST
EN
RT
gtZfL2-2
Amp

(B)

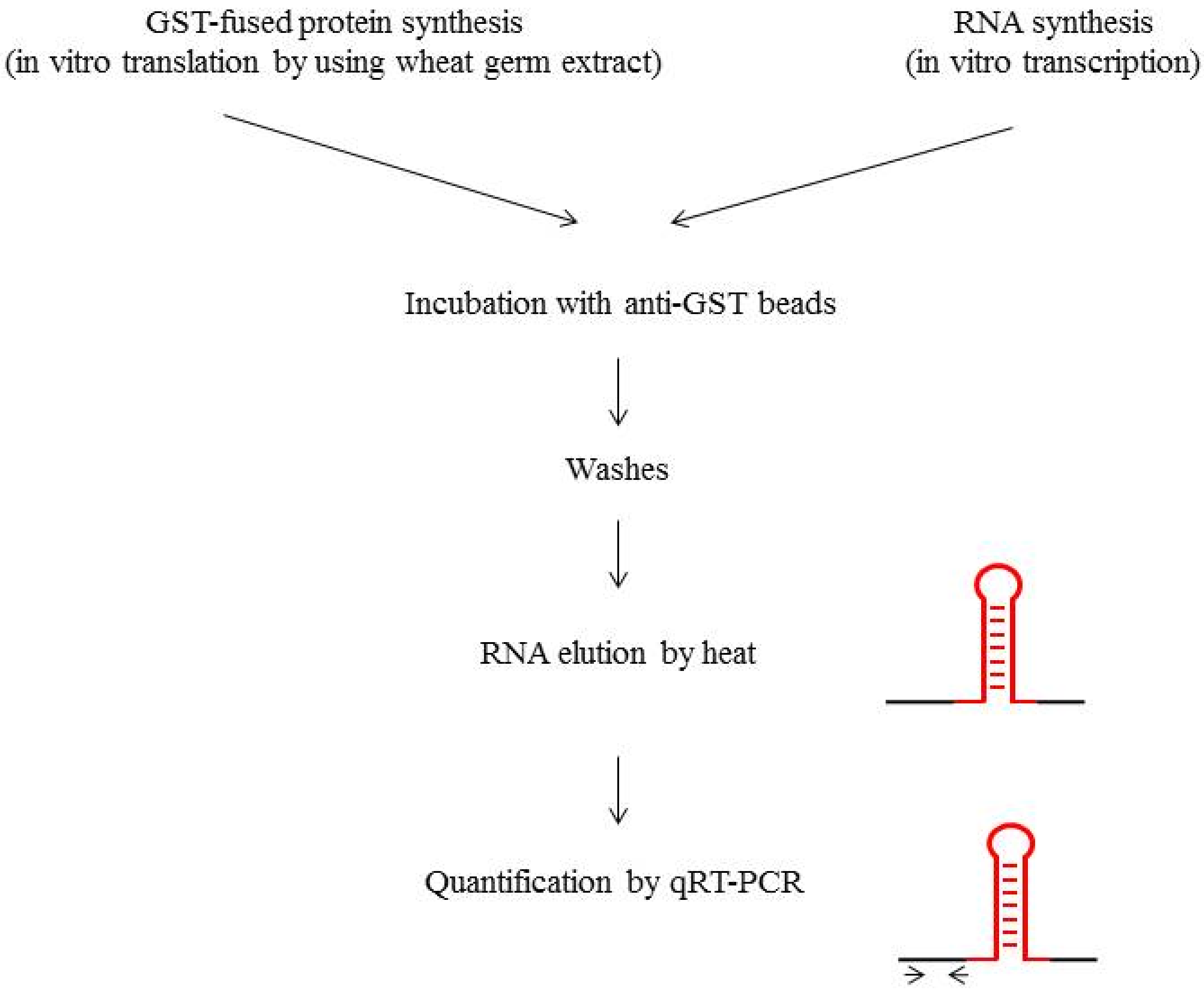
GST-fused protein synthesis
(in vitro translation by using wheat germ extract)
RNA synthesis
(in vitro transcription)
Incubation with anti-GST beads
Washes
RNA elution by heat
Quantification by qRT-PCR

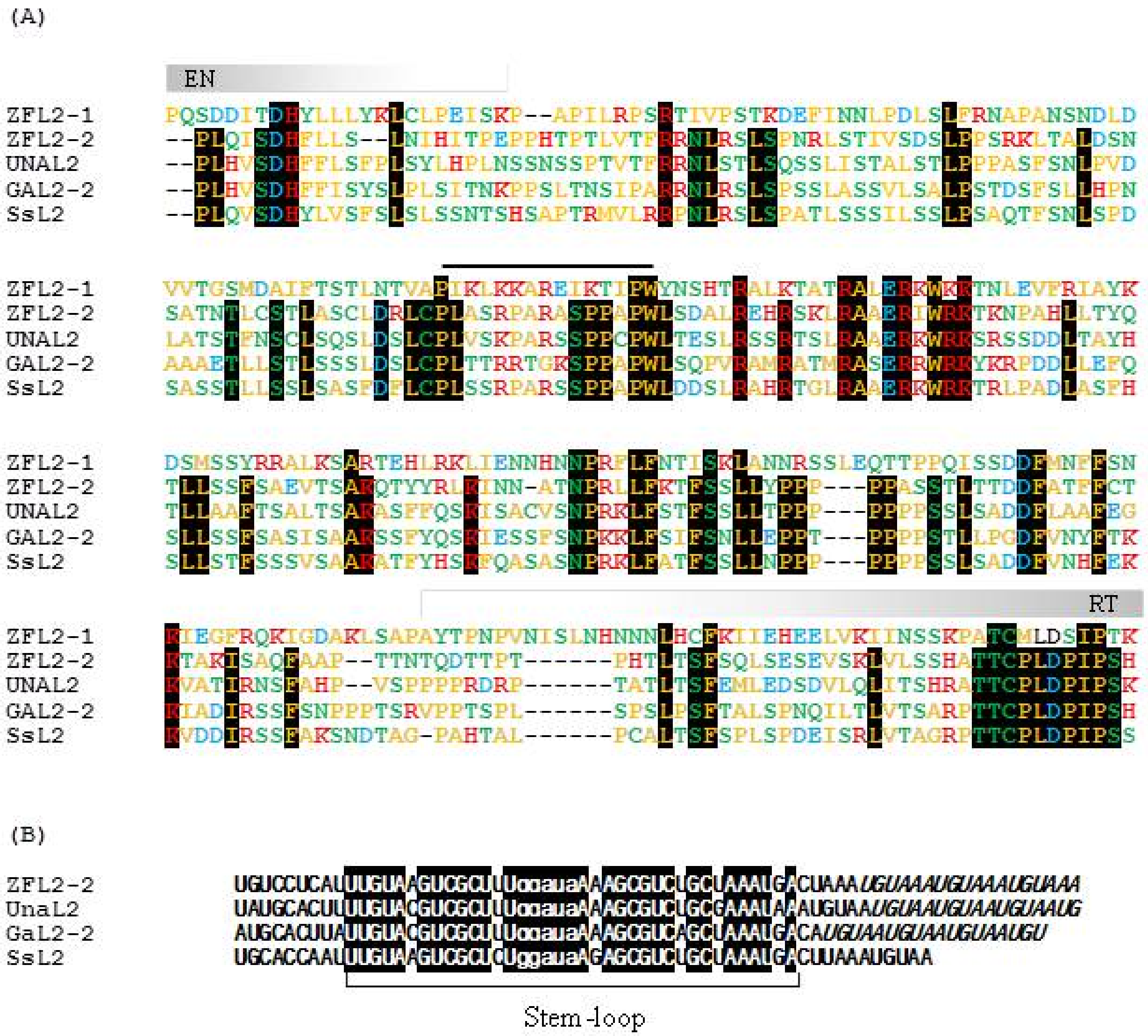
(A)
EN
ZFL2-1 PQSDDITDHYLLLYKLCLPEISKP--APILRPSRTIVPSTKDEFINNLPDLSLFRNAPANSNDLD
ZFL2-2 --PLQISDHFLLS--LNIHITPEPPHTPTLVTFRRNLRSLSPNRLSTIVSDSLPPSRKLTALDSN
UNAL2 --PLHVSDHFFLSFPLSYLHPLNSSNSSPTVTFRRNLSTLSQSSLISTALSTLPPPASFSNLPVD
GAL2-2 --PLHVSDHFFISYSLPLSITNKPPSLTNSIPARRNLRSLSPSSLASSVLSALPSTDSFSLLHPN
SsL2 --PLQVSDHYLVSFSLSLSSNTSHSAPTRMVLRRPNLRSLSPATLSSSILSSLPSAQTFSNLSPD
ZFL2-1 VVTGSMDAIFTSTLNTVAPIKLKKAREIKTIPWYNSHTRALKTATRALERKWKRTNLEVFRIAYK
ZFL2-2 SATNTLCSTLASCLDRLCPLASRPARASPPAPWLSDALREHRSKLRAAERIWRRTKNPAHLLTYQ
UNAL2 LATSTFNSCLSQSLDSLCPLVSKPARSSPPCPWLTESLRSSRTSLRAAERKWRRSRSSDDLTAYH
GAL2-2 AAAETLLSTLSSSLDSLCPLTTRRTGKSPPAPWLSQPVRAMRATMRASERRWRRYKRPDDLLEFQ
SsL2 SASSTLLSSLSASFDFLCPLSSRPARSSPPAPWLDDSLRAHRTGLRAAERKWRRTRLPADLASFH
ZFL2-1 DSMSSYRRALKSARTEHLRKLIENNHNNPRFLFNTISKLANNRSSLEQTTPPQISSDDFMNFFSN
ZFL2-2 TLLSSFSAEVTSAKQTYYRLKINN-ATNPRLLFKTFSSLLYPPP---PPASSTLTTDDFATFFCT
UNAL2 TLLAAFTSALTSAKASFFQSKISACVSNPRKLFSTFSSLLTPPP---PPPPSSLSADDFLAAFEG
GAL2-2 SLLSSFSASISAAKSSFYQSKIESSFSNPKKLFSIFSNLLEPPT---PPPPSTLLPGDFVNYFTK
SsL2 SLLSTFSSSVSAAKATFYHSKFQASASNPRKLFATFSSLLNPPP---PPPPSSLSADDFVNHFEK
RT
ZFL2-1 KIEGFRQKIGDAKLSAPAYTPNPVNISLNHNNNLHCFKIIEHEELVKIINSSKPATCMLDSIPTK
ZFL2-2 KTAKISAQFAAP--TTNTQDTTPT------PHTLTSFSQLSESEVSKLVLSSHATTCPLDPIPSH
UNAL2 KVATIRNSFAHP--VSPPPPRDRP------TATLTSFEMLEDSDVLQLITSHRATTCPLDPIPSK
GAL2-2 KIADIRSSFSNPPPTSRVPPTSPL------SPSLPSFTALSPNQILTLVTSARPTTCPLDPIPSH
SsL2 KVDDIRSSFAKSNDTAG-PAHTAL------PCALTSFSPLSPDEISRLVTAGRPTTCPLDPIPSS
(B)
ZFL2-2 UGUCCUCAUUUGUAAGUCGCUUUccauaAAAGCGUCUGCUAAAUGACUAAAUGUAAAUGUAAAUGUAAA
UnaL2 UAUGCACUUUUGUACGUCGCUUUccauaAAAGCGUCUGCGAAAUAAAUGUAAUGUAAUGUAAUGUAAUG
GaL2-2 AUGCACUUAUUGUACGUCGCUUUccauaAAAGCGUCAGCUAAAUGACAUGUAAUGUAAUGUAAUGU
SsL2 UGCACCAAUUUGUAAGUCGCUCUggauaAGAGCGUCUGCUAAAUGACUUAAAUGUAA
Stem-loop

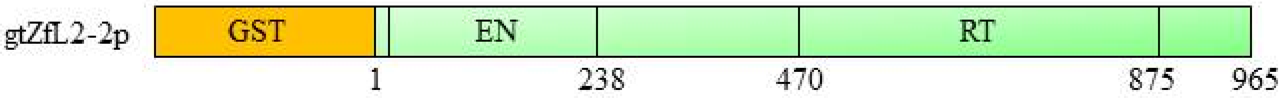

(a) gtZfL2-2(R338AA339E)p

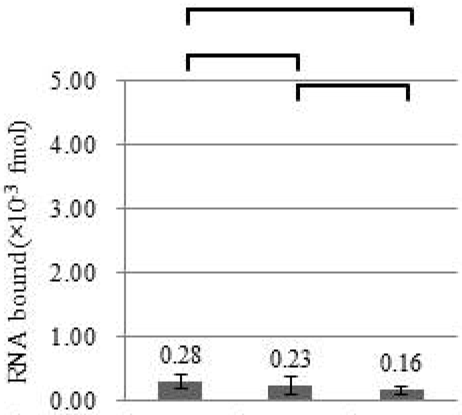

RNA	NoSL	SL1WT	SL2WT
n=	3	3	3

(b) gtZfL2-2(F415SF416S)p

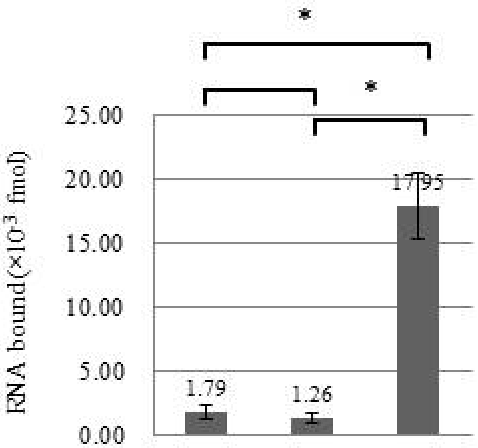

RNA	NoSL	SL1WT	SL2WT
n=	5	5	5

Supplementary Figure-5(Okada)

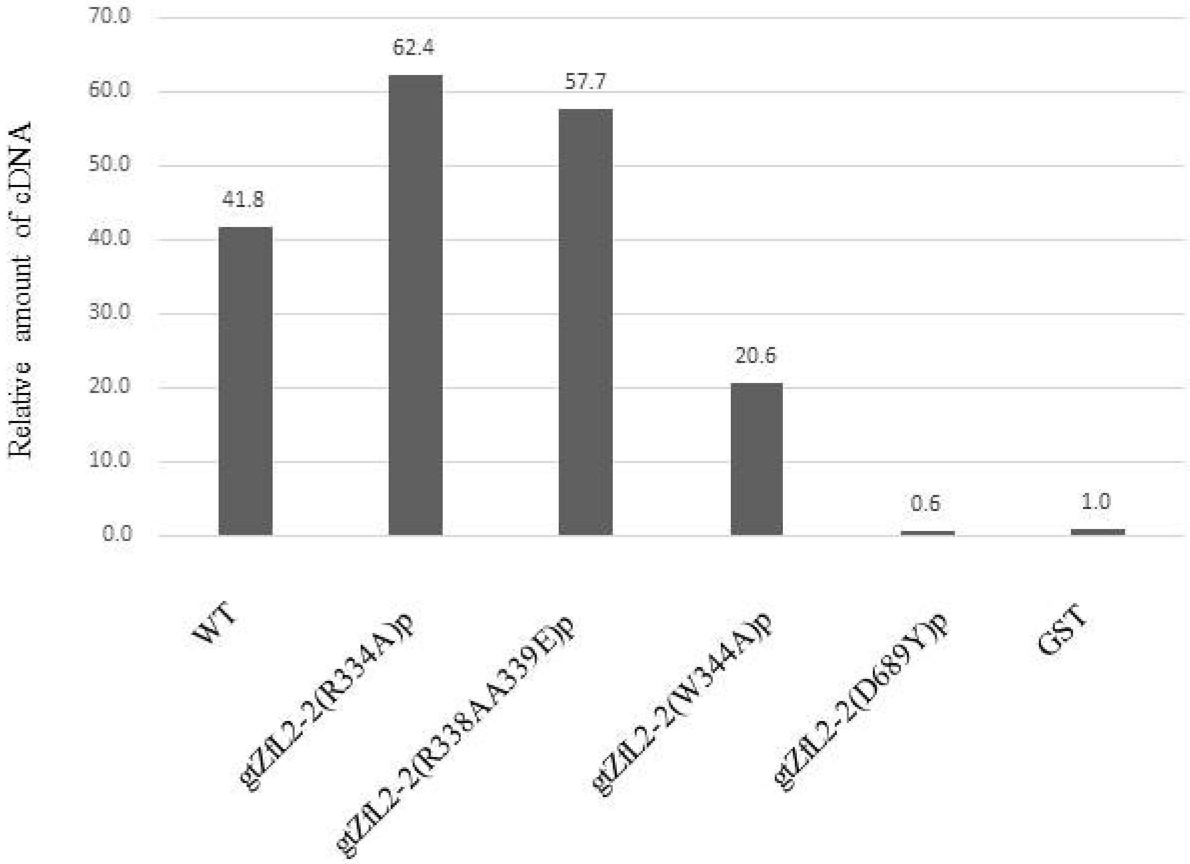

Supplementary Figure-6(Okada)

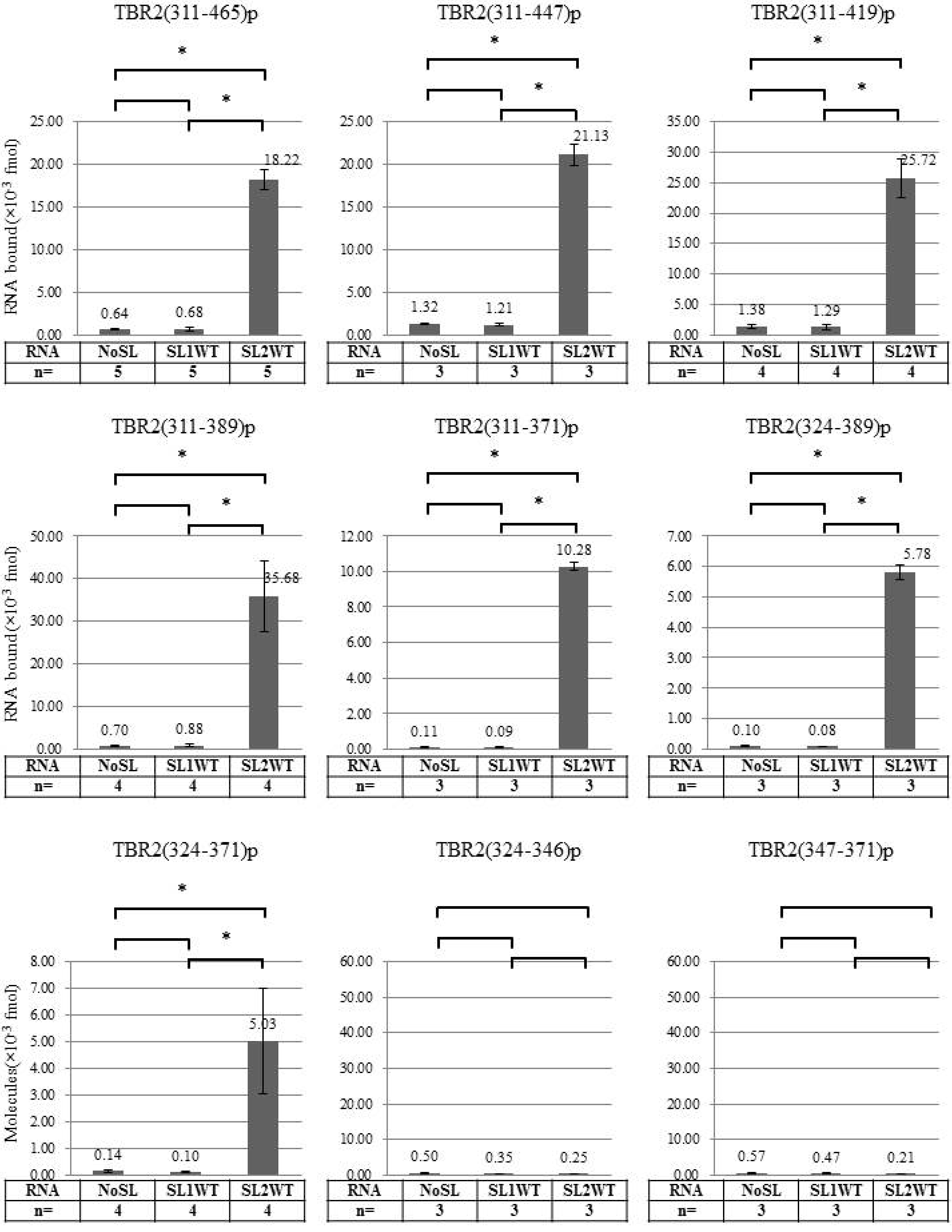

Supplementary Figure-7(Okada)

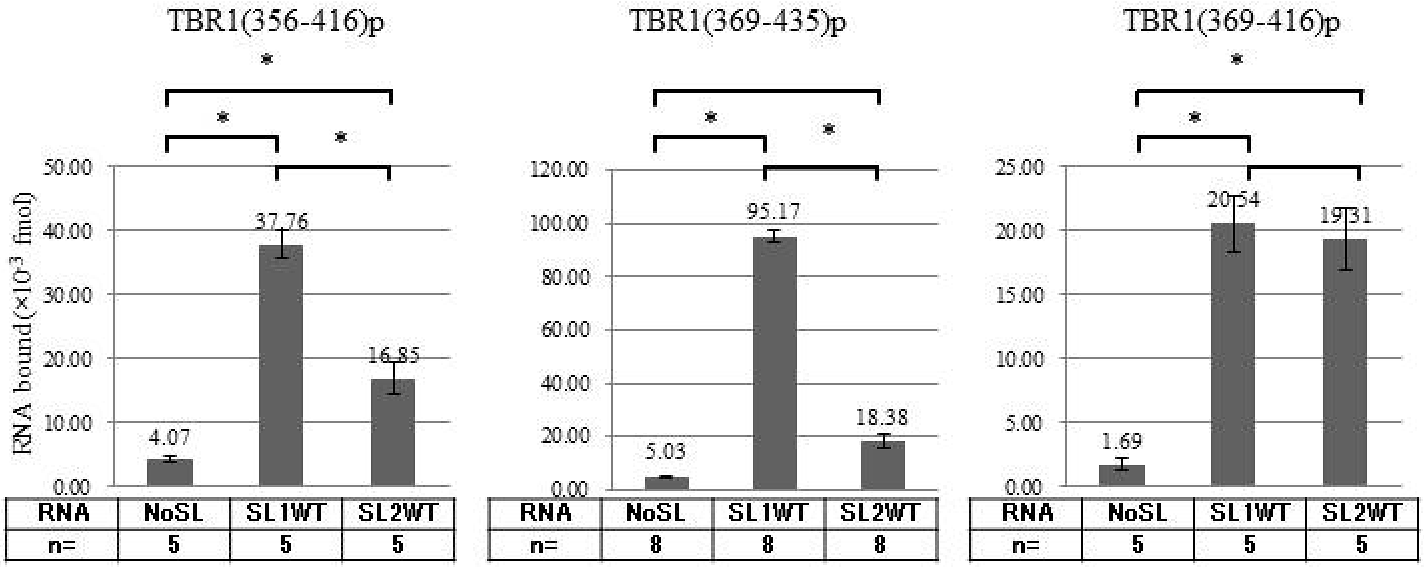

TX1(R376A)p

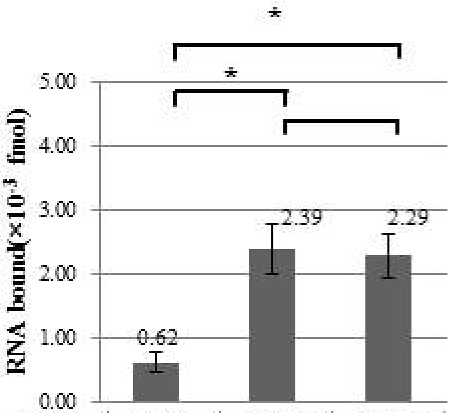

RNA	NoSL	SL1WT	SL2WT
n=	6	6	6

TX1(K379A)p

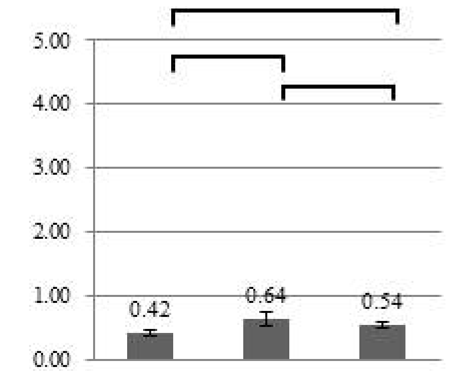

RNA	NoSL	SL1WT	SL2WT
n=	3	3	3

TX1(R383A)p

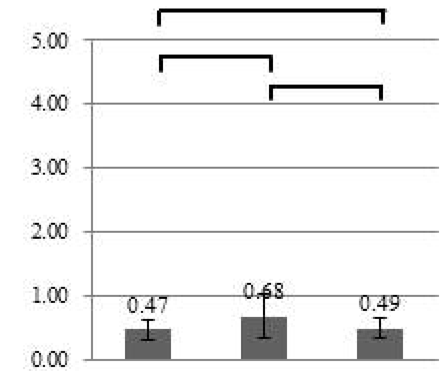

RNA	NoSL	SL1WT	SL2WT
n=	3	3	3

TX1(R387A)p

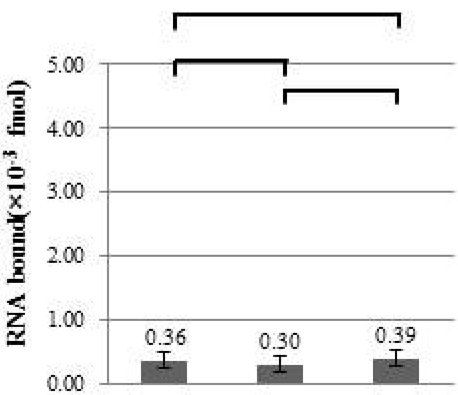

RNA	NoSL	SL1WT	SL2WT
n=	3	3	3

TX1(K390A)p

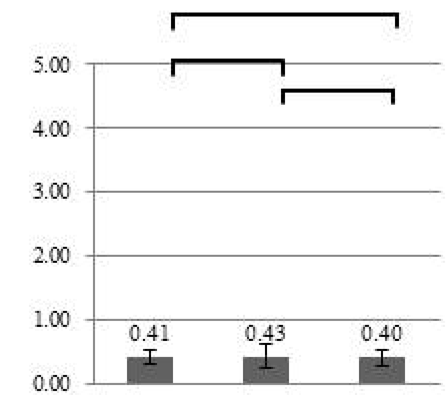

RNA	NoSL	SL1WT	SL2WT
n=	3	3	3

TX1(K391A)p

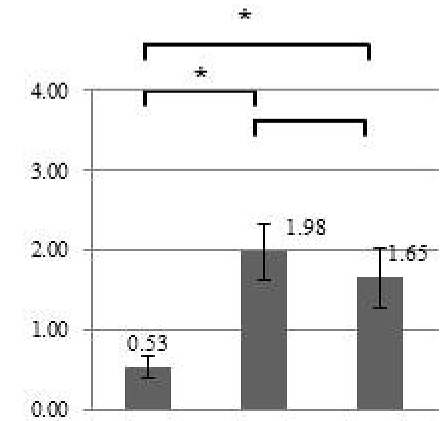

RNA	NoSL	SL1WT	SL2WT
n=	7	7	7

TX1(R398L)p

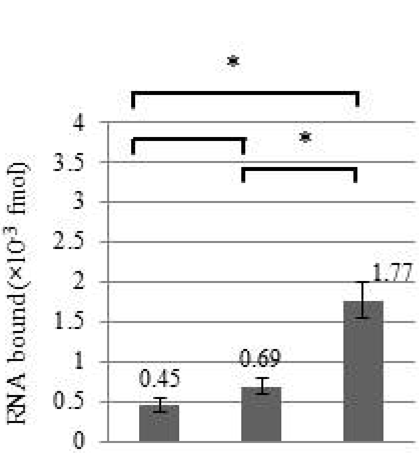

RNA	NoSL	SL1WT	SL2WT
n=	7	7	7

TX1(K402Q)p

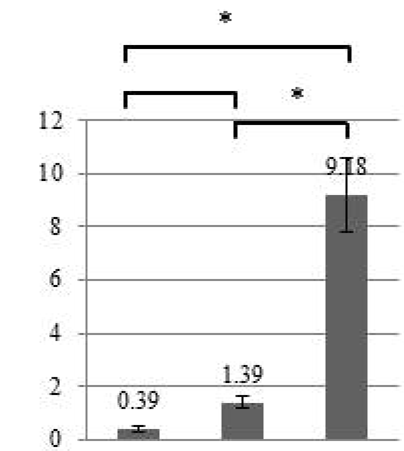

RNA	NoSL	SL1WT	SL2WT
n=	7	7	7

TX1(R409S)p

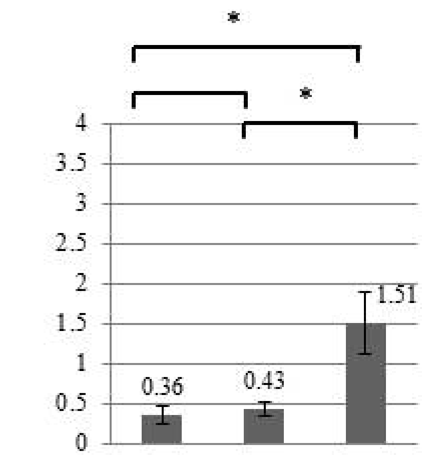

RNA	NoSL	SL1WT	SL2WT
n=	8	8	8

TX1(R410A)p

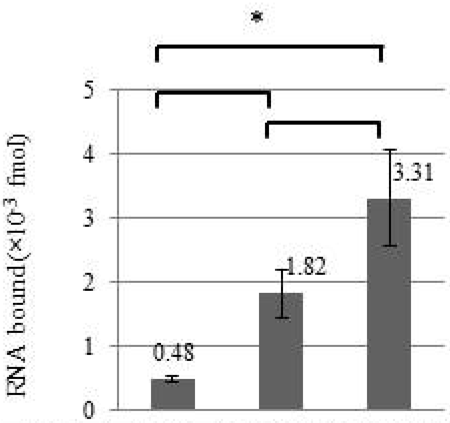

RNA	NoSL	SL1WT	SL2WT
n=	7	7	7

TX1(K413T)p

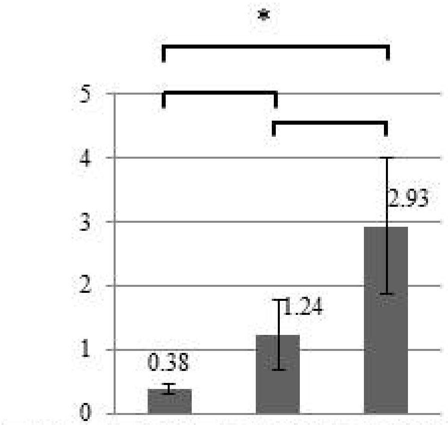

RNA	NoSL	SL1WT	SL2WT
n=	7	7	7

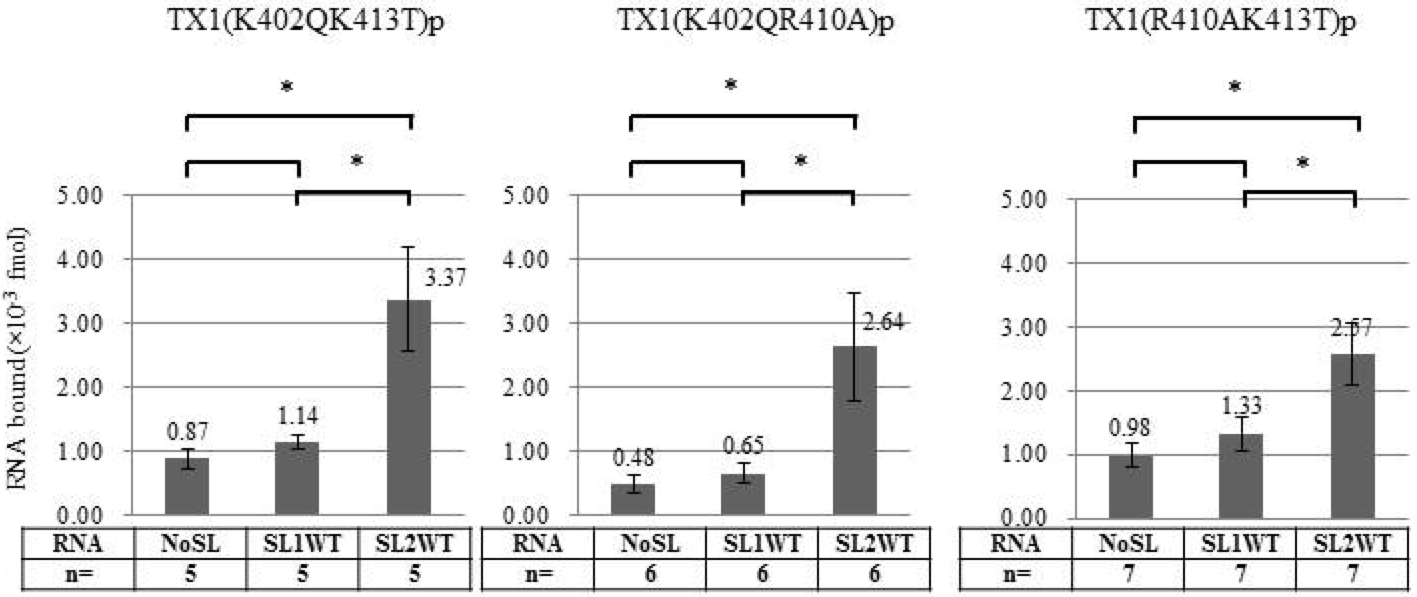

RNA	NoSL	SL1WT	SL2WT
n=	5	5	5

RNA	NoSL	SL1WT	SL2WT
n=	6	6	6

RNA	NoSL	SL1WT	SL2WT
n=	7	7	7

Z2SL2 Z2ΔSL2 Z2m(W325A) Z2m(R334A)

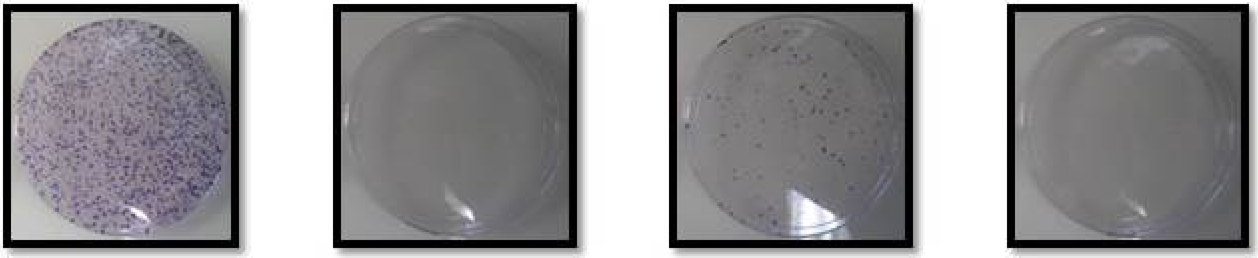

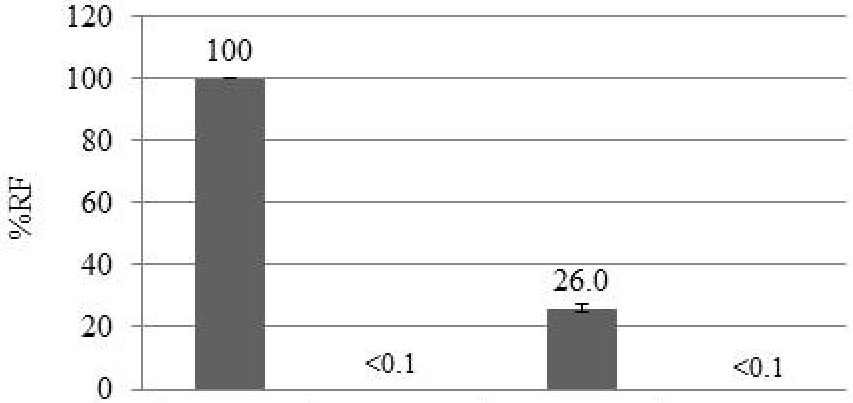

LINE	Z2SL2	Z2ΔSL2	Z2m(W325A)	Z2m(R334a)
n=	3	3	3	3
